# FTO suppresses glycolysis and growth of papillary thyroid cancer via decreasing stability of APOE mRNA in an N6-methyladenosine-dependent manner

**DOI:** 10.1186/s13046-022-02254-z

**Published:** 2022-01-28

**Authors:** Jiapeng Huang, Wei Sun, Zhihong Wang, Chengzhou Lv, Ting Zhang, Dalin Zhang, Wenwu Dong, Liang Shao, Liang He, Xiaoyu Ji, Ping Zhang, Hao Zhang

**Affiliations:** Department of Thyroid Surgery, The First Hospital of China Medical University, No. 155 in Nanjing North Street, Heping Distinct, Shenyang, Liaoning Province China

**Keywords:** Papillary thyroid cancer (PTC), N6-methyladenosine (m^6^A), FTO, APOE, Glycolysis

## Abstract

**Background:**

N6-methyladenosine (m^6^A) modification is the most common chemical modification in mammalian mRNAs, and it plays important roles by regulating several cellular processes. Previous studies report that m^6^A is implicated in modulating tumorigenesis and progression. However, dysregulation of m^6^A modification and effect of m^6^A demethylase fat-mass and obesity-associated protein (FTO) on glucose metabolism has not been fully elucidated in papillary thyroid cancer (PTC).

**Methods:**

Quantitative real-time PCR (qRT-PCR), western blotting and immunohistochemistry were performed to explore the expression profile of FTO in PTC tissues and adjacent non-cancerous thyroid tissues. Effects of FTO on PTC glycolysis and growth were investigated through in vitro and in vivo experiments. Mechanism of FTO-mediated m^6^A modification was explored through transcriptome-sequencing (RNA-seq), methylated RNA immunoprecipitation sequencing (MeRIP-seq), MeRIP-qPCR, luciferase reporter assays, RNA stability assay and RNA immunoprecipitation assay.

**Results:**

FTO expression was significantly downregulated in PTC tissues. Functional analysis showed that FTO inhibited PTC glycolysis and growth. Further analyses were conducted to explore FTO-mediated m^6^A modification profile in PTC cells and Apolipoprotein E (APOE) was identified as the target gene for FTO-mediated m^6^A modification using RNA-seq and MeRIP-seq. FTO knockdown significantly increased APOE mRNA m^6^A modification and upregulated its expression. FTO-mediated m^6^A modification of APOE mRNA was recognized and stabilized by the m^6^A reader IGF2BP2. The findings showed that APOE also promoted tumor growth through glycolysis in PTC. Analysis showed that FTO/APOE axis inhibits PTC glycolysis by modulating IL-6/JAK2/STAT3 signaling pathway.

**Conclusion:**

FTO acts as a tumor suppressor to inhibit tumor glycolysis in PTC. The findings of the current study showed that FTO inhibited expression of APOE through IGF2BP2-mediated m^6^A modification and may inhibit glycolytic metabolism in PTC by modulating IL-6/JAK2/STAT3 signaling pathway, thus abrogating tumor growth.

**Supplementary Information:**

The online version contains supplementary material available at 10.1186/s13046-022-02254-z.

## Background

Thyroid cancer is the most prevalent malignant tumor of the endocrine system, and its global incidence has been increasing significantly over the past decades [1]. The global cancer statistics in 2020 ranked thyroid cancer incidence as the 9th leading cancer cases among malignant tumors. Global incidence of thyroid cancer in women is three times higher compared with the incidence in men, and one in every 20 women diagnosed with malignancy has thyroid cancer [1]. Incidence of thyroid cancer in China is approximately twice the world average, and is the 7th leading cause of malignant tumors in the country [2]. Approximately 95% of the pathological types of thyroid cancer are composed of differentiated thyroid cancer (DTC), and the most common pathological subtype of DTC is papillary thyroid cancer (PTC) [3]. Most PTC cases have a good prognosis after standard surgery, TSH suppression and radioiodine therapy. However, approximately 30% patients present with recurrence and distant metastasis, thus significantly decreasing the survival rate [4, 5]. Moreover, occurrence and progression of PTC are affected by several factors such as genetic mutations, environmental exposure, and epigenetic alterations [6]. However, the specific molecular mechanism for occurrence and development of PTC has not been fully elucidated. Therefore, there is need to explore the molecular mechanism underlying occurrence and development of PTC, and to identify new diagnostic and therapeutic targets for PTC.

N6-methyladenosine (m^6^A) modification is the most common internal chemical modification in higher eukaryotic RNA, and it mainly modifies messenger RNA (mRNA) at the post-transcriptional level [7, 8]. m^6^A modification occurs in approximately 25% of transcripts at the genome-wide level. Its sites are mainly enriched near the stop codons, 5′- and 3′-untranslated regions, and within long internal exons [9–11]. Each transcript contains approximately 3–5 m^6^A sites, accounting for about 0.1–0.4% of the adenosine nucleotides [12]. The most frequent consensus sequence of m^6^A is RRACH (R = A/G/U, A = m^6^A, H = A/C/U) [10]. m^6^A is regulated by RNA methyltransferase (writers) that contain methyltransferase-like 3 (METTL3), methyltransferase-like 14 (METTL14) and Wilms tumor 1 associated protein (WTAP) in mammalian cells. The methylation can be reversed dynamically by the action of demethylases (erasers) including alkylation repair homolog protein 5 (ALKBH5) and fat-mass and obesity-associated protein (FTO). Notably, m^6^A is recognized and bound by m^6^A-binding proteins (readers) such as YTH domain family proteins 1–3 (YTHDF1–3) [7, 13], YTH domain-containing proteins 1–2 (YTHDC1–2) [14, 15] and insulin-like growth factor 2 mRNA-binding proteins 1–3 (IGF2BP1–3) [16]. m^6^A modification is involved in all stages of RNA life cycle and plays a role in regulating pre-mRNA processing [17], mRNA stability [7], translation efficiency [8], and nuclear export [18]. Recent studies report that m^6^A modification plays an important role in regulating initiation and progression of various cancers [19–22]. However, the role of m^6^A modification and the underlying regulatory mechanism in PTC has not been fully explored.

Reprogramming energy metabolism is a hallmark of cancer. Energy metabolism is reprogrammed to maintain cancer cells survival and logarithmic growth. Cancer cells reprogram their glucose metabolism even under aerobic conditions, utilize glycolysis instead of mitochondrial oxidative phosphorylation, which is termed aerobic glycolysis [23]. This non-canonical metabolic phenomenon is known as the Warburg effect [24]. Aerobic glycolysis of cancer cells consumes more glucose owing to its lower efficiency of ATP production and produces lactic acid. Studies have explored reprogramming of energy metabolism, and report that targeting glycolysis is a promising therapeutic strategy [25]. Aberrant expression of Bcl-2-associated athanogene 3 (BAG3) can reprogram glucose metabolism in pancreatic ductal adenocarcinoma, thus increasing expression of Hexokinase II (HK-II) which is the first key enzyme of glycolysis by inducing IMP3 recruitment [26]. However, the detailed role and mechanism of glycolysis in PTC has not been elucidated. Moreover, recent studies report that aerobic glycolysis of cancer cells is accompanied by dysregulated lipid metabolism, and some key factors of lipid metabolism may play a role in glycolysis of cells [27]. Apolipoprotein E (APOE) is the main component of plasma lipoproteins responsible for cholesterol transport and metabolism [28]. Although the role of APOE in cholesterol homeostasis and its regulatory mechanism in atherosclerosis and Alzheimerʼs disease (AD) are well known, the role and mechanism of APOE in cancer remains elusive [29]. APOE expression is significantly upregulated in gastric cancer and is correlated with shorter survival. In addition, APOE upregulation is positive correlated with muscular invasion, thus it is a potential biomarker for predicting extent of invasion [30]. However, expression profile and function of APOE in PTC is still unclear.

The current study explored the role of m^6^A modification in PTC. The anti-oncogene role of demethylases FTO and its relationship with clinicopathological features in PTC as then explored. Mechanistic studies showed that FTO regulates glycolysis and growth of PTC, which is dependent on m^6^A modification of APOE. Further analysis showed that FTO exerts its activity by modulation the IL-6/JAK2/STAT3 signaling pathway. The findings of the current study provide new insight into the potential mechanism of m^6^A modification in modulating glycolysis and growth of PTC.

## Material and methods

### Human specimens and cell lines

A total of 150 paired PTC tissue samples and their adjacent non-cancerous thyroid tissue samples were obtained from the Thyroid Surgery Department of the First Hospital of China Medical University between 2017 and 2018. One hundred pairs of PTC tissues and adjacent non-cancerous tissues (Cohort 1) were used in quantitative real-time reverse transcription PCR (qRT-PCR), 30 pairs (Cohort 2) were used for immunohistochemistry and 20 pairs (Cohort 3) were used for western blotting. Immunohistochemistry was performed on formalin-fixed and paraffin-embedded (FFPE) tissue specimens, while qRT-PCR and western blotting were performed on fresh frozen tissue specimens. All tissue specimens were independently confirmed by two pathologists. Written informed consent was obtained from all study participants. This study was approved by the Ethics Committee of the First Hospital of China Medical University. Human thyroid follicular epithelial cell line Nthy-ori3–1 and PTC cell lines including TPC1, K1, IHH4 and BCPAP were used in the current study. The source and culture method of these cell lines are described in additional file 2: supplementary materials and methods.

### Cell transfection and lentivirus infection

FTO, APOE, IGF2BP1, IGF2BP2, IGF2BP3 and NC siRNAs were obtained from Gene Pharma (Suzhou, China), their sequences are presented in Additional file 1 (Table S2). Transfections were performed using Lipofectamine 3000 (Invitrogen, Waltham, MA, USA) according to the manufacturer’s instructions. Recombinant lentiviruses containing sh-FTO or sh-APOE were constructed to stably knockdown FTO or APOE stably. Non-targeting shRNA (sh-NC) was used as negative control. FTO or APOE were overexpressed using recombinant lentiviruses containing complete coding sequences of these genes. In addition, we cloned the amplified mutant FTO-CDS from pcDNA3.1_mutFTO (carrying H231A and D233A, which disrupt the demethylase activity of FTO) into lentivector-based pMIRNA1 according to the methods of Li et al. [31] and Jia et al. [32]. Empty lentivirus (Vector) was used as negative control. All lentivirus vectors were constructed by Obio Technology (Shanghai, China). Infected cells were selected by puromycin. Transfection and infection efficiency were determined using qRT-PCR and western blotting.

### Seahorse metabolic analysis

Extra cellular acidification rate (ECAR) and oxygen consumption rate (OCR) were determined using Seahorse XF Glycolysis Stress Test Kit and Seahorse XF Cell Mito Stress Test Kit (Agilent Technologies, Palo Alto, CA), respectively. Transfected PTC cells were seeded into Seahorse dedicated 96-well cell culture plates overnight. Glucose, oligomycin and 2-deoxy-glucose (2-DG) were sequentially added to the corresponding well on the sensor cartridge for ECAR measurement. Oligomycin, FCCP and Antimycin A & Rotenone were sequentially added to the corresponding well for OCR determination. The prepared cell plate was then analyzed by Seahorse XFe96 Analyzer and data were analyzed using Seahorse Wave software.

### Glucose uptake and lactate production

Glucose uptake was determined using Glucose Colorimetric Assay Kit (BioVision, USA) according to the manufacturer’s protocols. Transfected PTC cells were seeded into 6-well plates and incubated for 48 h. Glucose enzyme mix specifically oxidizes glucose, to generate a product which reacts with a dye to generate color (OD 570 nm). Glucose content in the cell culture medium was determined and cells in each well were counted to normalize glucose concentration. Glucose uptake was determined indirectly by determining the remained glucose content in cell culture medium.

Lactate production was determined using Lactate Colorimetric Assay Kit (BioVision, USA) according to the manufacturer’s protocols. Lactate specifically reacts with an enzyme mix to generate a product, which interacts with lactate probe to produce color (OD 570 nm). Lactate production in the cell culture medium was detected and cells in each well were counted to normalize lactate concentration.

### Luciferase reporter assay

Fragments from APOE containing the wild-type m^6^A motifs (APOE-Wt) and mutant m^6^A motifs (m^6^A was replaced by C, APOE-Mut) were subcloned into pMIR-REPORT firefly luciferase vector (Obio Technology, Shanghai, China). Wild type and mutant APOE luciferase plasmids were co-transfected with empty, sh-FTO and FTO plasmids, using Renilla luciferase expressing vector pRL-TK (Promega, Madison, WI, USA). Relative luciferase activity was determined using dual-luciferase reporter assay system (Promega, Madison, USA). Relative firefly luciferase activity normalized to Renilla luciferase activity was then determined.

### Transcriptome-sequencing (RNA-seq) and methylated RNA immunoprecipitation sequencing (MeRIP-seq)

RNA-seq and MeRIP-seq were performed by Cloud-Seq Biotech (Shanghai, China). rRNAs were eliminated from the total RNA before performing RNA-seq and RNA libraries were constructed. Quality control and quantification of the library was conducted and RNA-seq was performed on Illumina HiSeq instrument using 150 bp paired-ended mode. For m^6^A-sequencing, MeRIP was performed using GenSeqTM m^6^A-MeRIP Kit (GenSeq, China) following the manufacturer’s instructions. Both the input samples without immunoprecipitation and the m^6^A IP samples were used for construction of the RNA-seq library. m^6^A-sequencing was performed on Illumina HiSeq instrument using 150 bp paired-ended mode after evaluating quality of the library. We extracted the sequences of the first 1000 peaks with the largest enrichment multiples for each group (50 bp on both sides of the vertex) from MeRIP-seq data and then scanned them with dreme software to identify meaningful motif sequences [10, 33].

### MeRIP assays

Total RNA was extracted by RNAiso Plus (Takara, Japan), and DNAse was added to remove DNA. MeRIP assays were performed using the m^6^A RNA Enrichment Kit (Epigentek, USA) according to the manufacturer’s protocols. Target m^6^A-containing fragments were pulled down using a beads-bound m^6^A capture antibody, and RNA sequences in both ends of the m^6^A-containing regions were cleaved using Cleavage Enzyme Mix. The enriched RNA was then released, purified and eluted. QRT-PCR was performed following MeRIP to quantify changes in target gene m^6^A methylation.

### RNA immunoprecipitation (RIP) assays

RNA immunoprecipitation (RIP) assays were conducted using Magna RIP Kit (Millipore, New Bedford, MA) according to the manufacturer’s protocols. Cells were lysed with appropriate amount of complete RIP Lysis Buffer. RNA-binding protein were immunoprecipitated using anti-IGF2BP2 antibody (Proteintech, USA) and normal rabbit IgG. The co-precipitated RNAs were purified and dissolved in RNase-free water. Binding RNA targets were analyzed using qRT-PCR.

### Nude mice xenograft model and ^18^F-FDG PET imaging

Five weeks old female athymic BALB/c nude mice were purchased from Beijing HFK bioscience Co., Ltd. (Beijing, China) and used to construct the xenograft tumor model. Transfected K1 or TPC1 cells were injected into the subcutaneous tissue of the mouse. After 4 weeks, the mice were euthanized, and tumors were excised and weighed. Tumor volume was calculated using the formula: Tumor volume (mm^3^) = longer diameter × shorter diameter^2^/2. Mice were fasted for 8 h and administered with approximately 200-300 μCi of ^18^F-FDG through the lateral tail vein after being anesthetized by 1% pentobarbital sodium a day before they were euthanized. Mice were maintained in a cage at room temperature for 1 h, then placed in the prone position on the examination bed for micro-PET and micro-CT imaging. ^18^F-FDG uptake was quantified by drawing region of interest (ROI) using Metis Viewer software and plotting maximum standardized uptake values (SUVmax). All animal experiments were conducted in accordance with the principles and procedures outlined in the guidelines of the Institutional Animal Care and Use Committee of China Medical University.

### Statistical analysis

All statistical analyses were performed using SPSS 19.0 software (IBM, Chicago, USA) and GraphPad Prism 8.3.0 software (San Diego, USA). All experiments were performed in triplicates, and data were presented as mean ± standard deviation (SD). Comparisons between groups were performed using Student’s t test or analysis of variance. Differences in relative expression levels of FTO and APOE in PTC tissues and adjacent non-cancerous tissues were analyzed using Wilcoxon signed-rank test. Correlation between FTO or APOE expression levels and the patients’ clinicopathological characteristics were analyzed using chi-squared test. Statistical significance was indicated by *p*-values < 0.05. Raw sequencing data were uploaded to Gene Expression Omnibus (GEO) database with the accession number GSE181047.

Detailed materials and methods are presented in Additional file 2: supplementary materials and methods.

## Results

### Abnormal m^6^A modification is observed and FTO is downregulated in PTC

**Fig. 1:**
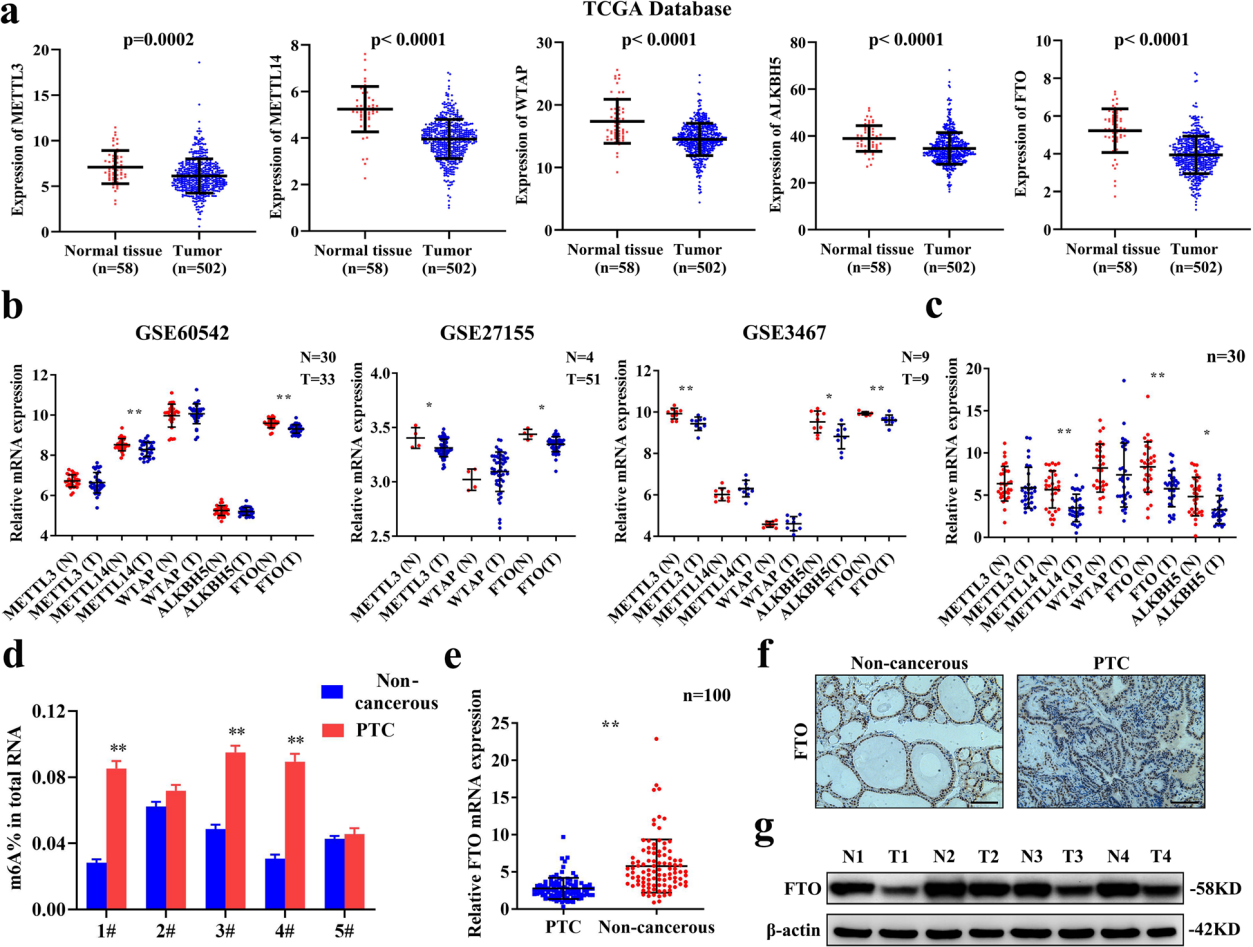
FTO is downregulated and the total m^6^A level was increased in PTC. **a-b** Expression level of m^6^A modification enzymes in TCGA and GEO database (GSE60542, GSE27155 and GSE3467). **c** Relative expression levels of m^6^A modification enzymes in 30 pairs PTC tissues and paired non-cancerous tissues as determined by qRT-PCR. **d** Total m^6^A levels in 5 pairs PTC samples as determined by m^6^A RNA Methylation Quantification Kit. **e** Relative expression levels of FTO in 100 pairs of PTC tissues and adjacent non-cancerous tissues as determined by qRT-PCR. **f** Representative photographs of IHC staining for FTO protein expression in PTC tissues and adjacent non-cancerous tissues (Cohort 2). Scale bars: 50 μm (400X). **g** Representative photographs of western blotting for FTO protein expression in PTC tissues (T) and paired non-cancerous tissues (N) (Cohort 3). *P < 0.05, **P < 0.01

To explore the role of the m^6^A modification in PTC, differential expression of m^6^A modification enzymes was determined in PTC tissues and normal thyroid tissues using public clinical databases including The Cancer Genome Atlas (TCGA) and GEO. Analysis using TCGA dataset showed that mRNA of the methylases METTL3, METTL14 and WTAP, as well as the demethylases FTO and ALKBH5 were significantly downregulated in PTC tissues compared with the expression level in normal tissues (Fig. 1a). In addition, the findings showed that expression of FTO was significantly downregulated in the 3 GEO datasets (GSE60542, GSE27155 and GSE3467) of PTC tissues compared with the expression in normal tissues (Fig. 1b). Expression level of m^6^A modification enzymes in the 30 pairs of PTC tissue samples in the current study was determined by qRT-PCR and the findings showed that the mRNA expression of FTO was downregulated in the PTC tissues (Fig. 1c). Consistent with our findings, the overall level of m^6^A was higher in PTC tissues compared with the level in the paired non-cancerous tissues (Fig. 1d). To further explore downregulation of FTO in PTC tissues, the tissue sample size was increased to 100 pairs and qRT-PCR analysis showed that FTO mRNA was downregulated in PTC tissues compared with the expression level in paired non-cancerous tissues (Fig. 1e). Analysis of the relationship between FTO expression and clinicopathological features showed that expression of FTO was significantly negatively correlated with extrathyroidal extension (*p* = 0.004) and lymph node metastasis (*p* = 0.035) (Table 1). In addition, we found that FTO expression was significantly negatively correlated with tumor size (*p* = 0.031) in TCGA database (Additional file 1: Fig. S4). Further, protein expression of FTO was determined in 30 paired PTC tissue samples by immunohistochemistry. The findings showed that FTO protein level was significantly downregulated in PTC tissues and mainly localized in the nuclei of PTC cells (Fig. 1f). Moreover, analysis by western blotting showed downregulation of protein expression of FTO in PTC tissues (Fig. 1g). These findings indicate that FTO is significantly downregulated in PTC and may play a role in regulation of PTC progression through m^6^A modification.

**Table 1:** Correlation between FTO expression and clinicopathological features in patients with PTC (*n* = 100)

Characteristic	n	FTO		***P*** value
		High expression (%)	Low expression (%)	
Gender				
Male	33	14 (42.4)	19 (57.6)	0.229
Female	67	37 (55.2)	30 (44.8)	
Age (Years)				
< 55	41	25 (61.0)	16 (39.0)	0.096
≥ 55	59	26 (44.1)	33 (55.9)	
Tumor size (cm)				
< 2	47	28 (59.6)	19 (40.4)	0.106
≥ 2	53	23 (43.4)	30 (56.6)	
Extrathyroidal invasion				
Yes	33	10 (30.3)	23 (69.7)	0.004*
No	67	41 (61.2)	26 (38.8)	
Lymph node metastasis				
Yes	72	32 (44.4)	40 (55.6)	0.035*
No	28	19 (67.9)	9 (32.1)	
TNM stage				
I-II	85	45 (52.9)	40 (47.1)	0.355
III-IV	15	6 (40.0)	9 (60.0)	

*P < 0.05

### FTO inhibits tumor growth in PTC in vitro and in vivo

**Fig. 2:**
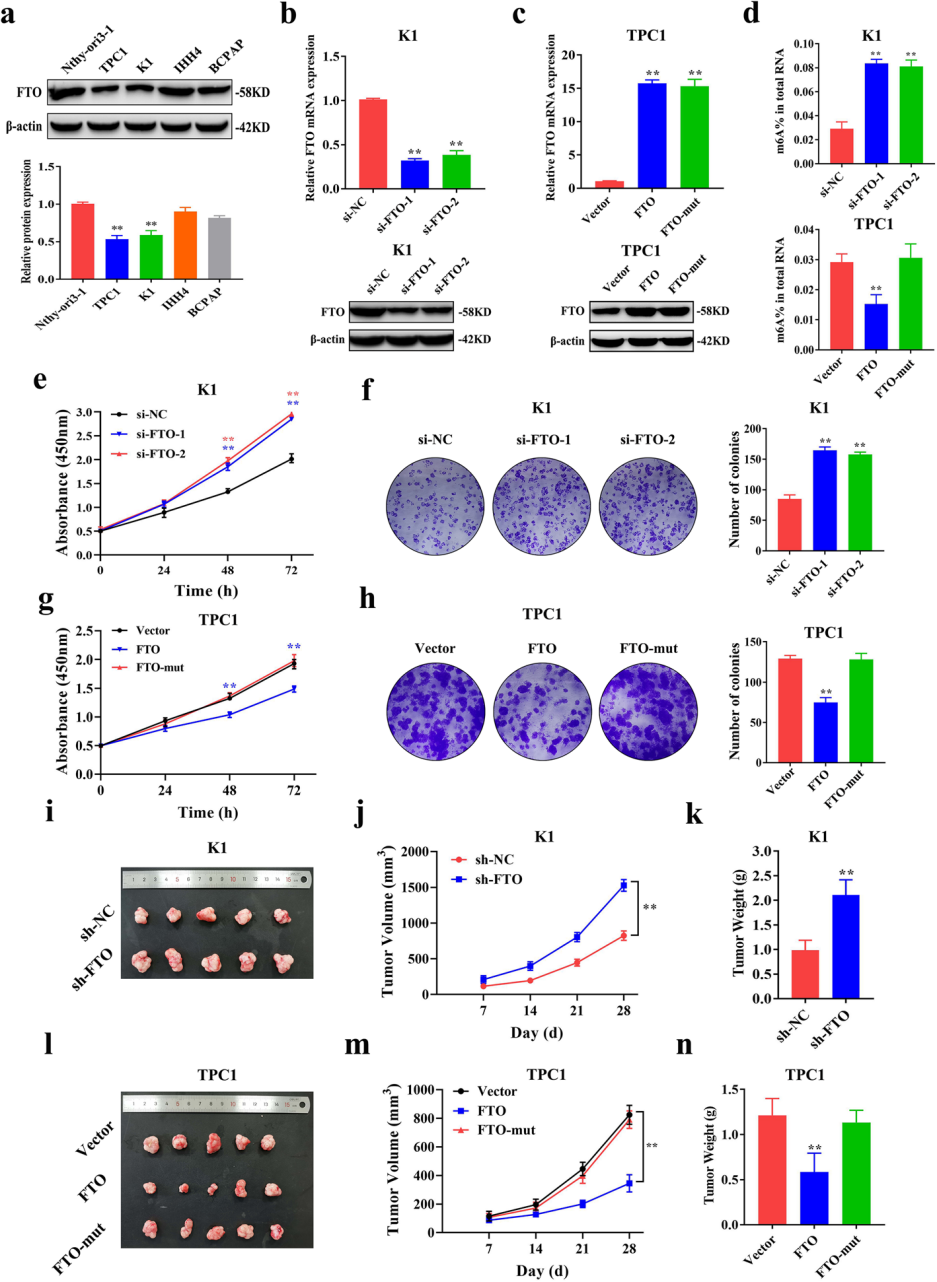
FTO inhibits PTC cell proliferation in vitro and in vivo. **a** Relative protein expression of FTO in the normal thyroid follicular epithelial cells and PTC cells as determined by western blotting. **b** Knockdown efficiency of si-FTO-1/2 in K1 cells as determined by qRT-PCR and western blotting. **c** Overexpression efficiency of FTO and FTO-mut in TPC1 cells as determined by qRT-PCR and western blotting. **d** Total m^6^A modification levels after FTO knockdown or overexpression in PTC cells as determined by m^6^A RNA Methylation Quantification Kit. **e-f** CCK-8 assay (**e**) and colony formation assay (**f**) showing proliferation ability in K1 cells after FTO knockdown. **g-h** CCK-8 assay (**g**) and colony formation assay (**h**) showing proliferation ability after FTO overexpression in TPC1 cells. **i** Representative image of subcutaneous tumors excised from nude mice model administered with K1 cells infected with sh-NC and sh-FTO (*n* = 5). **j-k** Tumor volumes (**j**) and tumor weights (**k**) were determined after excision from nude mice in the above-mentioned groups. **l** Representative image of subcutaneous tumors excised from nude mice model administered with TPC1 cells infected with empty vector, FTO and FTO-mut. **m-n** Tumor volumes (**m**) and tumor weights (**n**) were determined after excision from nude mice model in the above-mentioned groups. *P < 0.05, **P < 0.01

Relative expression of FTO was determined in a normal human thyroid follicular epithelial cell line Nthy-ori3–1 and four PTC cell lines (K1, BCPAP, IHH4, and TPC1). The findings showed that FTO expression was significantly lower in TPC1 and K1 cell lines compared with the level in the Nthy-ori3–1 cell line. However, FTO expression in IHH4 and BCPAP was slightly lower compared with the level in the Nthy-ori3–1 cell line (Fig. 2a). Therefore, KI and TPC1 cell lines which showed consistency with the expression trend of FTO in PTC tissues for subsequent experiments. Effect of FTO on cell proliferation in vitro and in vivo was explored to determine the role of FTO in PTC. Efficiency of FTO knockdown and overexpression of FTO was verified by qRT-PCR and western blotting (Fig. 2b-c and Additional file 3: Fig. S1a-b). The findings showed that knockdown or overexpression of FTO increased or decreased the global m^6^A modification level in PTC cells, respectively (Fig. 2d and Additional file 3: Fig. S1c). CCK-8 assays showed that FTO knockdown significantly promoted proliferation of PTC cells, and similar findings were obtained using colony formation assays (Fig. 2e, f and Additional file 3: Fig. S1f, g). Furthermore, it showed that overexpression of FTO inhibited PTC cell proliferation, whereas FTO-mut TPC1 cells with deletion of the demethylation domain showed no effect on proliferation (Fig. 2g, h and Additional file 3: Fig. S1d, e). These findings indicate that FTO-induced PTC proliferation depends on its demethylases function. In addition, we found that the proportion of cells decreased significantly in G0/G1 phase and increased significantly in S phase after FTO knockdown through cell cycle analysis, whereas FTO overexpression showed the opposite effects (Additional file 3: Fig. S1h, i). This result shows that FTO could block the transition of cell cycle from G0/G1 phase to S phase, thereby inhibiting its proliferation. Subsequently, we constructed sh-NC and sh-FTO stable cell lines and performed nude mice xenograft model experiments after confirming that FTO expression in sh-FTO group was significantly lower than that in sh-NC group (Additional file 3: Fig. S1j). Findings from in vivo experiments showed that knockdown of FTO significantly increased K1 tumor growth (Fig. 2i, j) and tumor weight (Fig. 2k) in xenograft mouse models. Furthermore, overexpression of FTO significantly reduced TPC1 tumor growth (Fig. 2l, m) and tumor weight (Fig. 2n) in xenograft mouse models. However, effect of FTO on tumor growth was not observed in vivo after overexpression of FTO-mut (Fig. 2l-n). These findings indicate that FTO inhibits tumor growth in PTC through its demethylation domain.

### FTO inhibits PTC growth by modulating glycolysis

**Fig. 3:**
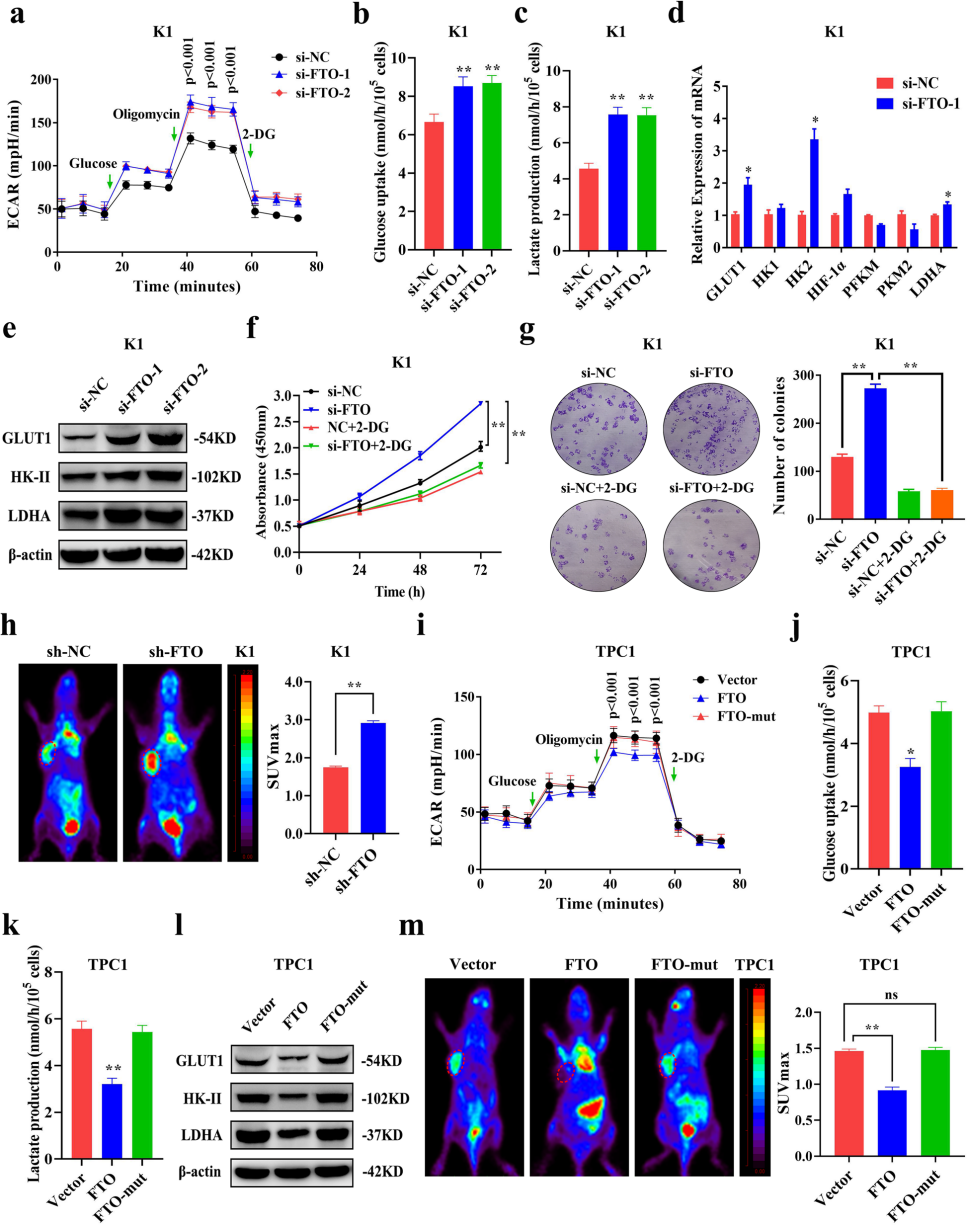
FTO inhibition of PTC growth by modulating glycolytic metabolism in vitro and in vivo. **a** Extra cellular acidification rate (ECAR) as determined by Seahorse metabolic analysis after transfection with si-NC and si-FTO in K1 cells. **b-c** Glucose uptake (**b**) and Lactate production (**c**) were determined after transfected with si-NC and si-FTO in K1 cells. **d** mRNA expression level of glycolytic enzymes was determined by qRT-PCR after FTO knockdown. **e** Protein expression level of GLUT1, HK-II and LDHA were determined by western blotting after FTO knockdown in K1 cells. **f-g** CCK-8 assay (**f**) and colony formation assay (**g**) showing proliferation ability after transfection with si-NC or si-FTO and simultaneous treatment with or without 2-Deoxyglucose (2-DG) in K1 cells. **h** Representative images of ^18^F-FDG uptake by micro-PET imaging in sh-NC and sh-FTO xenograft mouse models. Tumor glucose uptake is marked by red circles and maximum uptake values (SUVmax) for xenografts determined by FDG-PET are shown. **i** ECAR as determined by Seahorse metabolic analysis after FTO overexpression in TPC1 cells. **j-k** Glucose uptake (**j**) and Lactate production (**k**) were determined after FTO overexpression in TPC1 cells. **l** GLUT1, HK-II and LDHA protein expression levels as determined by western blotting after FTO overexpression in TPC1 cells. **m** Representative images of ^18^F-FDG uptake by micro-PET imaging in FTO overexpression xenograft mouse models. Tumor glucose uptake is indicated by red circles and maximum uptake values (SUVmax) for xenografts determined by FDG-PET are shown. *P < 0.05, **P < 0.01

Further analysis was conducted to explore the mechanism of action of FTO in modulating growth of PTC. FTO can maintain energy homeostasis by controlling energy consumption [34]. However, studies have not fully elucidated whether FTO plays a role in energy metabolism in PTC. To explore the regulatory effect of FTO on PTC energy metabolism, the effect of FTO on the glycolytic metabolism of PTC was determined through ECAR of Seahorse metabolic analysis. The findings showed that knockdown of FTO significantly increased ECAR levels in K1 and TPC1 cells (Fig. 3a and Additional file 3: Fig. S2a), implying that it can inhibit glycolysis in PTC. Moreover, analysis showed that OCR level was decreased after FTO knockdown, implying that it promoted mitochondrial oxidative phosphorylation in PTC, which also shows its inhibitory effect on glycolysis from the side (Additional file 3: Fig. S2b, c). Cancer cells increase glucose uptake to compensate for the lower efficiency of ATP produced by glycolysis compared with mitochondrial oxidative phosphorylation thus promoting uncontrolled cell growth and division. Glycolysis results in high amounts of lactate which can be utilized as the main energy source by other cells, and glycolytic intermediates may be diverted into various biosynthetic pathways [23]. Extracellular glucose analysis showed that glucose uptake was significantly increased after FTO knockdown in PTC cells (Fig. 3b and Additional file 3: Fig. S2d). Extracellular lactate level was determined as an indicator of glycolysis and the findings showed significantly increased lactate production in PTC cells after FTO knockdown (Fig. 3c and Additional file 3: Fig. S2e). The process of aerobic glycolysis involves several enzymes. Further analysis of regulatory mechanism of glycolysis was performed by determining expression levels of key enzymes in glycolysis after FTO knockdown. QRT-PCR analysis showed that GLUT1, HK-II and LDHA mRNA level were significantly upregulated after FTO knockdown in K1 cells (Fig. 3d). Western blotting showed that protein expression level of GLUT1, HK-II and LDHA were significantly upregulated after FTO knockdown (Fig. 3e and Additional file 3: Fig. S2f), further confirming the effect of FTO on glycolytic enzymes. PTC cells were treated with glycolysis inhibitor, 2-DG, and the findings showed that 2-DG inhibited proliferation and colony formation caused by FTO knockdown (Fig. 3f, g and Additional file 3: Fig. S2g, h). This finding indicated that FTO affects cell proliferation by modulating glycolysis. Further, effect of FTO on glycolysis in vivo was determined using ^18^F-FDG PET ([^18^F]-fluoro-2-deoxyglucose positron emission tomography) to determine glucose uptake in mice tumor. PET-CT findings showed that glucose uptake in xenograft mouse tumor model was significantly increased after FTO knockdown compared with the control (Fig. 3h).

Gain of function assays showed that overexpression of FTO significantly decreased ECAR levels, glucose uptake and lactate production in PTC cells (Fig. 3i-k and Additional file 3: Fig. S2i-k). Furthermore, OCR levels were significantly increased in PTC cells (Additional file 3: Fig. S2l, m). Moreover, GLUT1, HK-II and LDHA protein expression were significantly downregulated after FTO overexpression (Fig. 3l and Additional file 3: Fig. S2n). Overexpression of FTO significantly reduced glucose uptake in mice (Fig. 3m). However, the effect on glycolysis was not observed both in vitro and in vivo after overexpression of FTO-mut (Fig. 3i-m and Additional file 1: Fig. S2m), indicating that FTO modulates glycolysis through its demethylation domain. In summary, these findings indicate that FTO inhibits PTC progression by modulating glycolysis.

### RNA-seq and MeRIP-seq identified APOE as a direct target for FTO

**Fig. 4:**
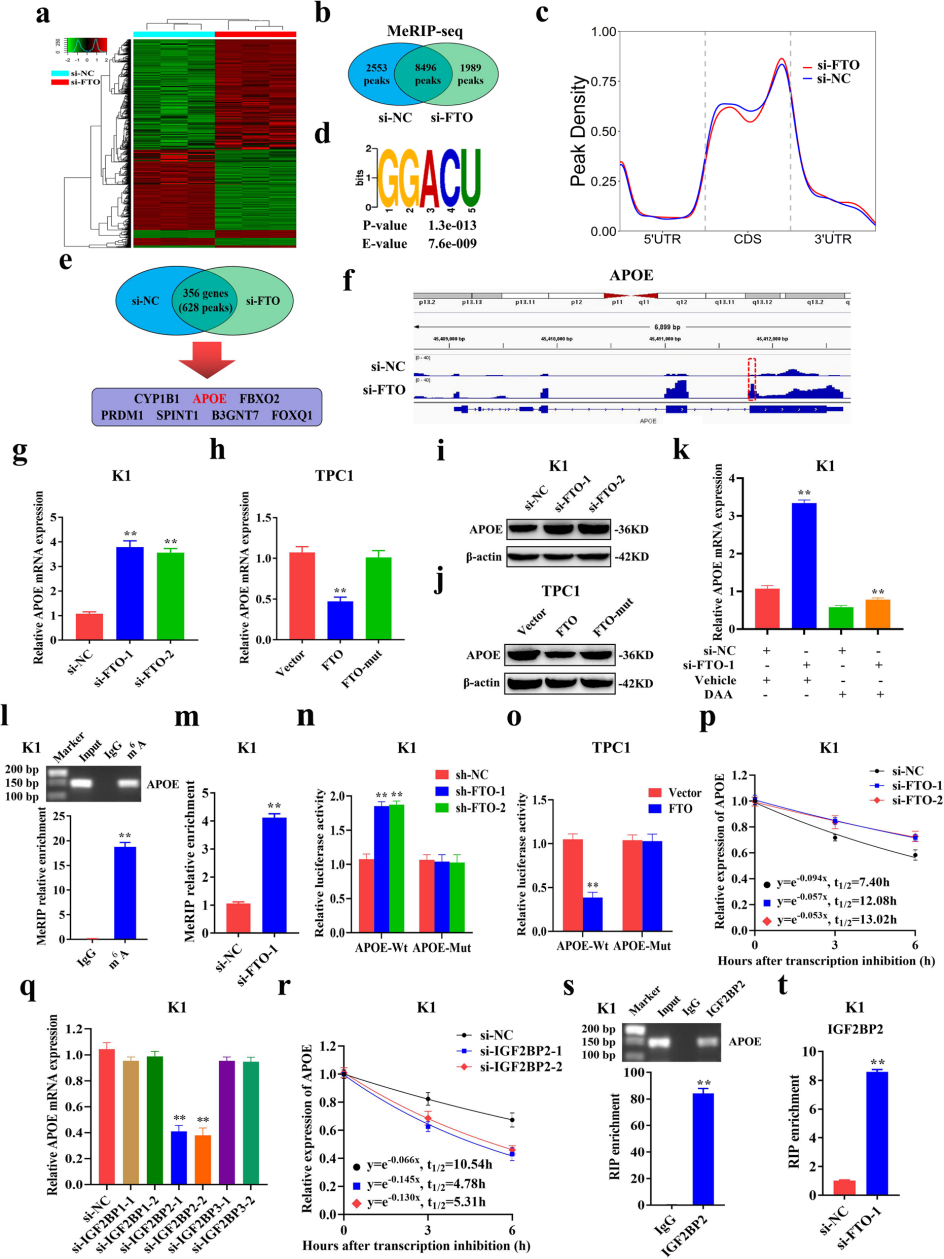
APOE mRNA is regulated by FTO-mediated m^6^A modification through the m^6^A reader IGF2BP2. **a** Differentially expressed genes between si-NC group and si-FTO group in K1 cells as determined by RNA-sequencing. **b** Peak profiles in m^6^A modification after FTO knockdown in K1 cells as shown by MeRIP-sequencing. **c** Distribution of m^6^A peaks in mRNA transcripts of K1 cells. **d** The m^6^A consensus motif present in K1 cells. **e** Differentially expressed genes accompanied with elevated m^6^A peaks after FTO knockdown were filtered and APOE was identified as the direct target of FTO. **f** m^6^A modification of APOE mRNA was visualized by Integrative Genomics Viewer (IGV) software after FTO knockdown. The significantly increased m^6^A peak is indicated by red rectangles. **g-j** APOE mRNA and protein expression level as determined by qRT-PCR and western blotting after FTO knockdown or overexpression in PTC cells. **k** Relative APOE mRNA expression as determined by qRT-PCR after transfection with si-NC or si-FTO and simultaneous treatment with or without 3-deazaadenosine (DAA, a global methylation inhibitor) in K1 cells. **l** Direct binding between m^6^A antibody and APOE mRNA was validated by agarose electrophoresis and MeRIP assays in K1 cells. **m** Relative enrichment of m^6^A antibody and APOE mRNA as determined by MeRIP-qPCR after FTO knockdown in K1 cells. **n-o** Relative luciferase activity of APOE-Wt and APOE-Mut as determined after FTO knockdown or overexpression in PTC cells. **p** Half-life (t_1/2_) of APOE mRNA as determined by qRT-PCR after FTO knockdown in K1 cells. **q** Relative expression of APOE mRNA as determined by qRT-PCR after IGF2BP1–3 knockdown in K1 cells. **r** Half-life (t_1/2_) of APOE mRNA as determined by qRT-PCR after IGF2BP2 knockdown in K1 cells. **s** Direct binding between IGF2BP2 protein and APOE mRNA as validated by agarose electrophoresis and RIP assays in K1 cells. **t** Relative enrichment of IGF2BP2 protein and APOE mRNA as determined by RIP-qPCR after FTO knockdown in K1 cells. *P < 0.05, **P < 0.01

To explore the downstream potential targets of FTO in PTC, RNA-seq was performed to determined differentially expressed genes (DEGs) of the si-NC group and si-FTO group using K1 cells. The findings showed that 490 genes were significantly downregulated, whereas 675 genes were significantly upregulated after FTO knockdown (Fig. 4a). To verify whether changes in gene expression were attributed to FTO-mediated m^6^A modification, MeRIP-seq was performed to identify significantly elevated m^6^A modification after FTO knockdown in K1 cells. MeRIP-seq findings showed 2553 unique m^6^A peaks in si-NC group, 1989 unique m^6^A peaks in si-FTO group and 8496 shared m^6^A peaks in both groups (Fig. 4b). Consistent with previous studies, analysis showed that m^6^A peaks were highly enriched around the stop codon in si-NC and si-FTO groups (Fig. 4c). Mapping of m^6^A methylomes in K1 cells showed that ‘GGACU’ which was highly enriched in the m^6^A peaks was the most common m^6^A motif (Fig. 4d). The average peak lengths of si-NC and si-FTO group are 1002.86 and 1037.31 respectively. Gene ontology (GO) analysis showed that the differentially expressed transcripts were significantly enriched in gene sets involved in glucose transmembrane transporter activity, indicating that FTO plays an important role in glycolysis (Additional file 3: Fig. S3a). Pathway analysis showed that ‘Pathways in cancer’ was significantly correlated with FTO-mediated m^6^A modification (Additional file 3: Fig. S3b). Then, we combined RNA-seq and MeRIP-seq data according to the items with the same gene name and screened out mRNAs that were differentially expressed and had significantly higher m^6^A modification levels following FTO knockdown according to the criteria of fold change > 2 and *p*-value < 1.0e-5. After obtaining 356 DEGs (353 upregulated genes and 3 downregulated genes) encompassing 628 increased m^6^A peaks from sequencing data, we queried their expression in PTC tissues and normal thyroid tissues using public databases such as TCGA database. Subsequently, we employed qRT-PCR to identify the expression of DEGs in PTC, which were predicted from the public database in our own paired PTC tissue samples. Finally, we identified seven genes containing 13 m^6^A peaks for further functional experiments and discovered that APOE might be the key gene for FTO-mediated m^6^A modification as it could modulate glycolytic metabolism of PTC. (Fig. 4e). Analysis of MeRIP-seq data using Integrative Genomics Viewer (IGV) software showed that APOE mRNA had one m^6^A peak, which was detected near the stop codon and was significantly higher after FTO knockdown (Fig. 4f). However, expression and biological function of APOE in PTC should be further elucidated. Therefore, APOE was selected as the optimal target for FTO-mediated m^6^A modification for subsequent analysis.

### FTO regulates APOE mRNA stability through m^6^A modification mediated by IGF2BP2

Consistent with sequencing analysis, the findings showed that knockdown or overexpression of FTO upregulated or downregulated expression of APOE at mRNA and protein levels (Fig. 4g-j and Additional file 3: Fig. S3c-f). However, APOE expression showed no difference after overexpression of FTO-mut (Fig. 4h, j). To explore m^6^A modification of APOE by FTO, 3-Deazaadenosine (DAA, an inhibitor of methylation) was used and the findings showed that DAA significantly decreased expression of APOE that was increased by FTO knockdown (Fig. 4k and Additional file 3: Fig. S3g). FTO-mediated m^6^A modification of APOE mRNA was preliminarily verified. To further explore this effect, m^6^A-seq data was validated by performing MeRIP-PCR. The findings showed that anti-m^6^A antibody significantly enriched APOE mRNA level in K1 cells (Fig. 4l). Moreover, MeRIP-qPCR showed that m^6^A levels of APOE mRNA were significantly increased after FTO knockdown (Fig. 4m). Subsequently, luciferase reporters containing either the wild-type or mutant APOE were constructed to explore the effect of m^6^A modification on APOE expression. Findings from luciferase reporter assays showed that luciferase activity of wild-type APOE was significantly increased after FTO knockdown whereas overexpression of APOE reduced luciferase activity. However, knocking down or overexpressing FTO showed no effect on the luciferase activity of mut-type APOE (Fig. 4n, o and Additional file 3: Fig. S3h, i). These findings indicate that FTO plays a role in m^6^A modification of APOE.

Expression of APOE increased significantly with increase in the level of m^6^A modification, implying that its m^6^A modification may increase stability of APOE mRNA [7]. Subsequently, RNA stability assays were conducted and RNA stability curves showed that half-life of APOE mRNA was significantly prolonged after FTO knockdown in K1 and TPC1 cells (Fig. 4p and Additional file 3: Fig. S3j). Therefore, upregulation of APOE induced by FTO can be attributed to increase in stability of APOE mRNA caused by m^6^A modification. Although m^6^A modification can be modulated by ‘writers’ and ‘erasers’, its functional outcomes are depended on selective recognition of m^6^A sites by ‘readers’. IGF2BP1–3 modulates stability of mRNA plays a role as a m^6^A reader [16]. Analysis showed that knocking down IGF2BP2, but not IGF2BP1/3 significantly downregulated APOE mRNA in K1 and TPC1 cells (Fig. 4q and Additional file 3: Fig. S3k). Moreover, half-life of APOE mRNA was significantly decreased after IGF2BP2 knockdown in K1 and TPC1 cells (Fig. 4r and Additional file 3: Fig. S3l). Analysis of the mechanism using RIP assay showed that IGF2BP2 directly bound to the m^6^A sites of APOE (Fig. 4s). Further qRT-PCR analysis for RIP assay showed that knockdown of FTO significantly increased binding efficiency of IGF2BP2 to APOE mRNA (Fig. 4t). In summary, these findings indicate that FTO inhibits APOE mRNA stability and expression through m^6^A modification mediated by IGF2BP2.

### APOE regulates proliferation in PTC by modulating glycolysis

**Fig. 5:**
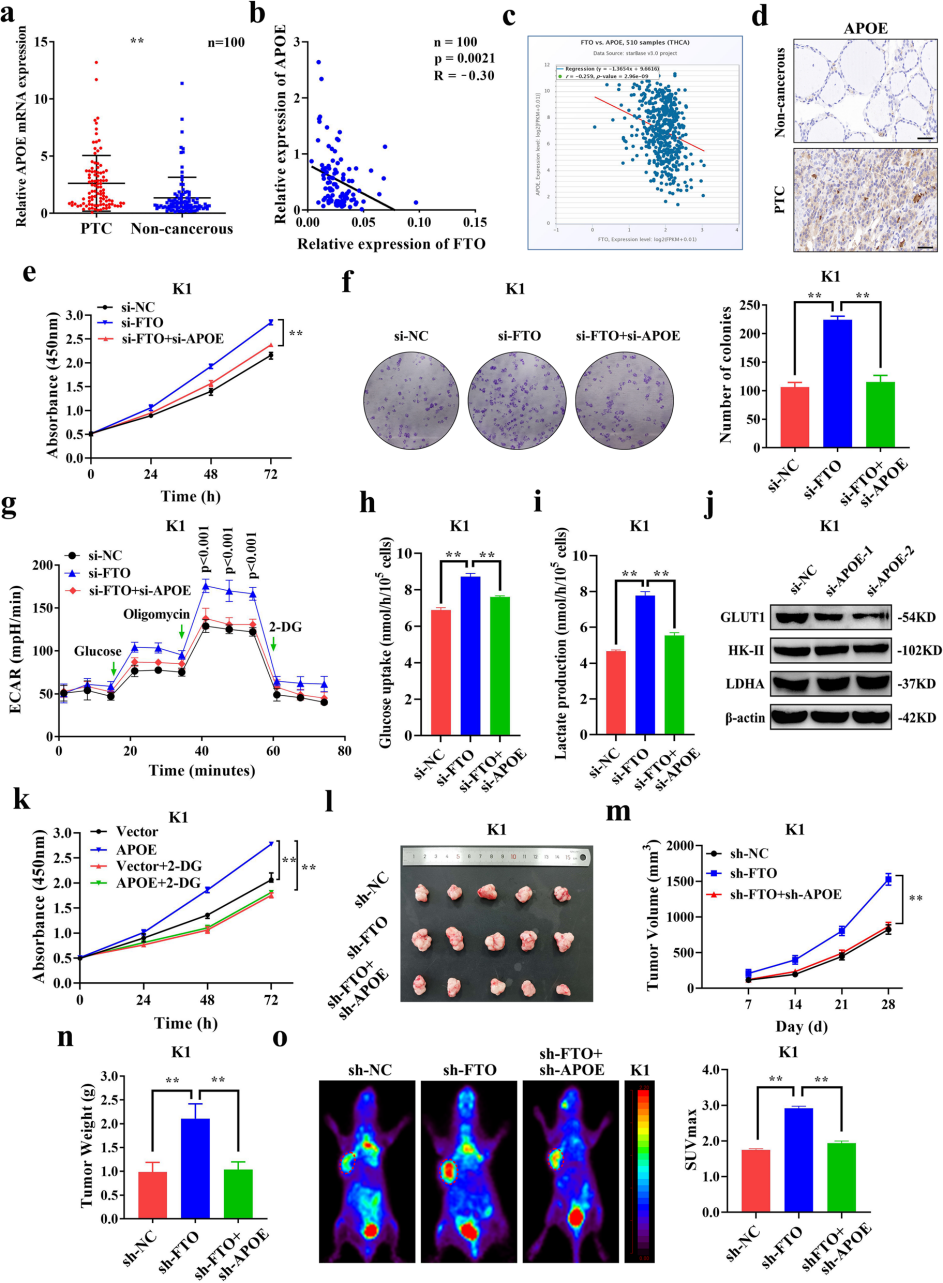
APOE is negatively correlated with FTO and regulates PTC proliferation by modulating glycolysis. **a** Relative expression levels of APOE as determined by qRT-PCR in 100 pairs of PTC tissues and adjacent non-cancerous tissues. **b** FTO is negatively correlated with APOE expression as shown in 100 pairs of PTC tissues. **c** FTO and APOE expressions showed a significantly negative correlation in TCGA database (Obtained from starBase). **d** Representative photographs of IHC staining showing APOE protein expression in PTC tissues and adjacent non-cancerous tissues (Cohort 2). Scale bars: 50 μm (400X). **e-f** CCK-8 assay (**e**) and colony formation assay (**f**) showing proliferation ability after transfection with si-FTO or co-transfected si-FTO and si-APOE in K1 cells. **g** ECAR as determined by Seahorse metabolic analysis after transfection with si-FTO or co-transfection with si-FTO and si-APOE in K1 cells. **h-i** Glucose uptake (**h**) and Lactate production (**i**) as determined after transfection with si-FTO or co-transfection with si-FTO and si-APOE in K1 cells. **j** Protein expression of GLUT1, HK-II and LDHA as determined by western blotting after APOE knockdown in K1 cells. **k** CCK-8 assay showing proliferation ability after transfection with empty vector or APOE and simultaneous treatment with or without 2-Deoxyglucose (2-DG) in K1 cells. **l** Representative image of subcutaneous tumors excised from nude mice model bearing K1 cells transfected with sh-NC, sh-FTO and sh-FTO+ sh-APOE (n = 5). **m-n** Tumor volumes (**m**) and tumor weights (**n**) determined after excision from nude mice in the above-mentioned groups. **o** Representative images of ^18^F-FDG uptake by micro-PET imaging in sh-NC, sh-FTO and sh-FTO+ sh-APOE xenograft mouse models. Tumor glucose uptake is indicated using red circles and maximum uptake values (SUVmax) for xenografts determined by FDG-PET are shown. *P < 0.05, **P < 0.01

Although the vital role of FTO in PTC growth by glycolysis were confirmed, it was unclear whether the effect was specifically attributed to APOE, the target gene for m^6^A modification downstream of FTO. To explore the role of APOE in PTC, expression level of APOE in cohort 1 was determined by qRT-PCR. The findings showed that mRNA expression of APOE was significantly upregulated in PTC tissues compared with the level in paired non-cancerous tissues (Fig. 5a). Analysis of the relationship between APOE expression and its clinicopathological features, showed that expression of APOE was significantly positively correlated with tumor size (*p* = 0.005) and extrathyroidal invasion (*p* = 0.028) (Table 2). In addition, we found that APOE expression was significantly negatively linked to age (*p* < 0.001), extrathyroidal invasion (*p* = 0.012) and TNM stage (*p* = 0.035) in TCGA database (Additional file 1: Fig. S5). In cohort 1, expression of APOE in PTC was significantly negatively correlated with expression level of FTO (Fig. 5b). Consistent with our results, the expression of FTO and APOE in TCGA database also showed a significantly negative correlation (R = -0.259, *p* = 2.96e-09) (Fig. 5c). In addition, protein expression of APOE was determined in PTC paired tissue samples of cohort 2. The findings showed that APOE protein expression was significantly higher in PTC tissues compared with adjacent non-cancerous tissues (Fig. 5d). Subsequently, the biological function of APOE in PTC growth was explored. Efficiency of APOE knockdown was confirmed by qRT-PCR and western blotting (Additional file 3: Fig. S4a-d). To explore whether APOE plays an important role as the target gene of FTO in PTC growth, rescue experiments were conducted to explore if APOE knockdown can reverse the effects of FTO knockdown. The findings showed that downregulation of APOE expression significantly reduced high proliferation levels induced by si-FTO (Fig. 5e, f and Additional file 3: Fig. S4e, f). Moreover, knocking down APOE significantly reduced the increased ECAR level induced by si-FTO, implying that APOE can reverse increase in glycolysis rate caused by FTO (Fig. 5g and Additional file 3: Fig. S4g). Moreover, the reduced OCR level induced by si-FTO was rescued by si-APOE, indicating the role of APOE in promoting glycolysis from another point of view (Additional file 3: Fig. S4h, i). In addition, extracellular glucose and lactate analysis showed that APOE knockdown reversed increased glucose uptake and lactate production induced by si-FTO (Fig. 5h, i and Additional file 3: Fig. S4j, k). Analysis of the effect of APOE on expression of key glycolysis proteins showed that only GLUT1 protein expression was significantly downregulated after APOE knockdown, whereas expression levels of HK-II and LDHA were not affected (Fig. 5j and Additional file 3: Fig. S4l). This finding indicates that FTO and APOE mainly affects glycolysis by regulating glucose transport. To explore whether APOE affects cell proliferation through glycolysis, PTC cells overexpressing APOE were treated with 2-DG. Similar to FTO, 2-DG inhibited the increased proliferation caused by overexpression of APOE (Fig. 5k and Additional file 3: Fig. S4m-o), indicating that APOE affects proliferation through glycolysis. In vivo analysis showed that sh-APOE significantly impaired tumor growth (Fig. 5l, m) and tumor weight (Fig. 5n) in xenograft mouse models caused by sh-FTO. Furthermore, micro-PET analysis showed that sh-APOE significantly reduced glucose uptake induced by sh-FTO in xenograft mouse models (Fig. 5o). These findings indicate that APOE could promote tumor growth through glycolysis and is regulated by FTO-mediated m^6^A modification in PTC.

**Table 2:** Correlation between APOE expression and clinicopathological features in patients with PTC (n = 100)

Characteristic	n	APOE		***P*** value
		High expression (%)	Low expression (%)	
Gender				
Male	33	18 (54.5)	15 (45.5)	0.619
Female	67	33 (49.3)	34 (50.7)	
Age (Years)				
< 55	41	19 (46.3)	22 (53.7)	0.437
≥ 55	59	32 (54.2)	27 (45.8)	
Tumor size (cm)				
< 2	47	17 (36.2)	30 (63.8)	0.005*
≥ 2	53	34 (64.2)	19 (35.8)	
Extrathyroidal invasion				
Yes	33	22 (66.7)	11 (33.3)	0.028*
No	67	29 (43.3)	38 (56.7)	
Lymph node metastasis				
Yes	72	40 (55.6)	32 (44.4)	0.144
No	28	11 (39.3)	17 (60.7)	
TNM stage				
I-II	85	43 (50.6)	42 (49.4)	0.854
III-IV	15	8 (53.3)	7 (46.7)	

*p < 0.05

### FTO and APOE may regulate glycolysis in PTC through IL-6/JAK2/STAT3 signaling pathway

**Fig. 6:**
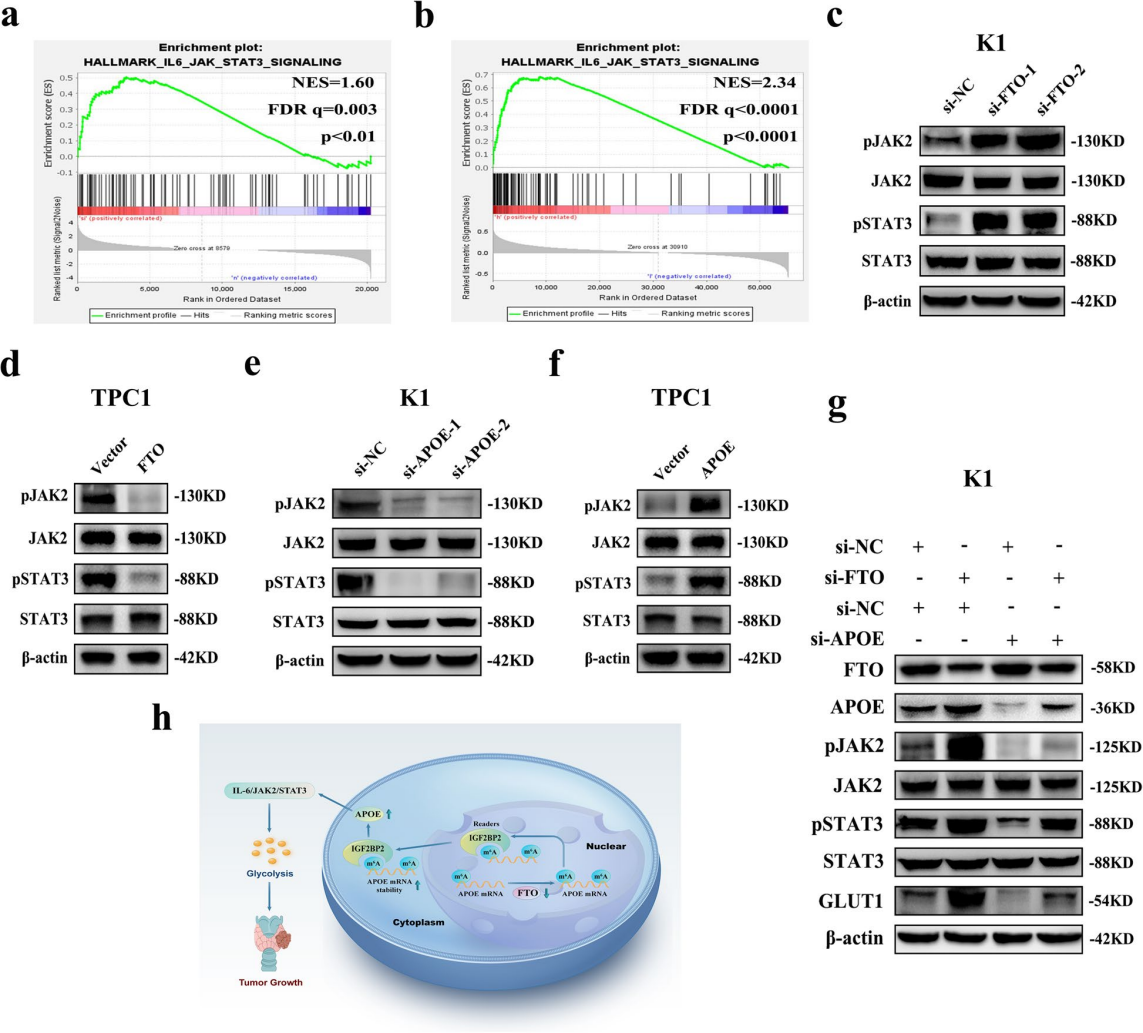
FTO and APOE regulate glycolysis of PTC by modulating IL-6/JAK2/STAT3 signaling pathway. **a** Differentially expressed gene profiles based on GSEA of si-FTO and si-NC RNA-seq data. **b** Differential gene profiles based on GSEA of APOE gene expression profile data of TCGA. **c** Expression levels of IL-6/JAK2/STAT3 signaling pathway related proteins as determined by western blotting after FTO knockdown in K1 cells. **d** Expression levels of IL-6/JAK2/STAT3 signaling pathway related proteins as determined by western blotting after FTO overexpression in TPC1 cells. **e** Expression levels of IL-6/JAK2/STAT3 signaling pathway related proteins as determined by western blotting after APOE knockdown in K1 cells. **f** Expression levels of IL-6/JAK2/STAT3 signaling pathway related proteins as determined by western blotting after APOE overexpression in TPC1 cells. **g** Expression levels of GLUT1 protein and IL-6/JAK2/STAT3 signaling pathway related proteins as determined by western blotting after co-transfection of si-FTO and si-APOE in K1 cells. **h.** Mechanism diagram of the relationship of FTO, m^6^A modification, PTC tumor growth and glycolysis metabolism. *P < 0.05, **P < 0.01

To explore the related signaling pathway downstream of FTO/APOE axis, Gene set enrichment analysis (GSEA) was performed to identify the enriched signature of FTO and APOE in PTC. GSEA analysis found that FTO and APOE have their own related signaling pathways, but the common signaling pathway of FTO/APOE downstream was only the IL-6/JAK/STAT3 signaling pathway. GSEA based on the RNA-seq data showed that IL-6/JAK/STAT3 signaling pathway was highly enriched in si-FTO group compared with the si-NC group (Fig. 6a). Moreover, GSEA based on transcriptome data of APOE expression in PTC from the TCGA database showed that IL-6/JAK/STAT3 signaling pathway was highly enriched in APOE high-expression group compared with the low-expression group (Fig. 6b). In addition, the APOE high-expression group were highly enriched in thyroid cancer (Additional file 3: Fig. S5a). These GSEA findings indicated that FTO and APOE may affect glycolysis of PTC by modulating IL6/JAK/STAT3 signaling pathway. Subsequently, effects of FTO and APOE on expression of related proteins in this pathway were explored by western blotting. The findings showed that the protein expression levels of phosphorylated JAK2 (pJAK2) and STAT3 (pSTAT3) in this pathway were significantly increased after FTO knockdown, whereas FTO overexpression showed the opposite effects. (Fig. 6c, d and Additional file 3: Fig. S5b, c). In addition, we found that the protein expression levels of these two phosphorylated protein were significantly decreased after APOE knockdown, whereas APOE overexpression showed the opposite effects. (Fig. 6e, f and Additional file 3: Fig. S5d, e). Furthermore, the findings showed that APOE knockdown significantly abrogated the increased pJAK2, pSTAT3 and GLUT1 protein expression level caused by si-FTO (Fig. 6g and Additional file 3: Fig. S5f). In summary, these findings indicate that APOE exerts its effect on glycolysis in PTC tissues modulating IL6/JAK2/STAT3 signaling pathway after m^6^A modification by FTO.

## Discussion

RNA m^6^A modification is a novel epigenetic modification which plays vital biological roles by regulating various cellular processes. Dysregulation of m^6^A modification is a potential pathogenesis mechanism in many diseases, mainly in cancers. Previous studies report that m^6^A modification regulators, such as “writers” (METTL3, METTL14 and WTAP) and “easers” (ALKBH5 and FTO) plays an indispensable role in regulating cell proliferation, metastasis, self-renewal, and resistance to chemotherapy in multiple cancers [19–21]. Therefore, several studies are exploring m^6^A modification to understand progression of cancers thus developing effective therapies. However, the potential involvement of m^6^A modification in human thyroid cancer has not been explored, particularly its role in modulating glycolysis metabolism in PTC. Previous studies report contradicting findings on the role of m^6^A modification regulators in different cancers. FTO is a m^6^A demethylase that promotes breast tumor progression by inhibiting BNIP3 [35], whereas another study reported inhibitory effect of FTO on metastasis by targeting demethylating metastasis-associated protein 1 (MTA1) mRNA in colorectal cancer [36]. However, in ovarian cancer, FTO acts as a tumor suppressor and regulates cyclic AMP signaling involved in stemness and tumor initiation [37]. In addition to the contradictory phenomena about the function of FTO in different cancers, the role of FTO in PTC has not been fully elucidated. The findings of the present study showed that FTO expression was significantly downregulated, and may be implicated in the abnormally increased m^6^A modification in PTC. Moreover, downregulation of FTO expression was significantly negatively correlated with extrathyroidal extension and lymph node metastasis. Notably, FTO knockdown significantly promoted glycolysis metabolism and proliferation in PTC cells, whereas overexpression of FTO inhibited glycolysis and proliferation of PTC cells. ^18^F-FDG PET scan analysis showed that FTO knockdown significantly promoted tumor growth and glucose uptake in xenograft mouse tumor model in vivo. These findings indicated that FTO plays a tumor suppressor role in PTC.

To further explore the role of FTO, MeRIP-seq and RNA-seq analysis was performed to determine whether APOE is the target of m^6^A modification downstream of FTO. The m^6^A modification site of APOE was located near the stop codon. APOE was negatively regulated by FTO and modified by FTO-mediated m^6^A methylation as validated through MeRIP-qPCR and luciferase reporter assays. Previous studies report that m^6^A modified mRNA transcripts are mainly mediated by m^6^A readers such as YTHDFs or IGF2BPs [7, 8, 13–16]. The mRNA transcripts with m^6^A modification are targeted by different m^6^A readers thus they may have opposite outcomes. YTHDC2, YTHDF2 and YTHDF3 downregulates expression of the target gene by promoting decay of their mRNA transcripts [38–40], whereas IGF2BP1–3 upregulates expression of target genes by promoting stability of their mRNA [41]. The findings of the current study showed that FTO knockdown significantly prolonged half-life of APOE mRNA and promoted its expression, indicating that the stability of APOE was significantly increased after m^6^A modification. Therefore, these findings imply that the m^6^A modification of APOE may be recognized and mediated by IGF2BPs. Further analysis showed that IGF2BP2 knockdown significantly reduced expression of APOE in PTC cells. Moreover, RNA stability assay showed that half-life of APOE mRNA was significantly reduced after IGF2BP2 knockdown, and findings from RIP assay indicated that IGF2BP2 binds to APOE mRNA. These findings indicate that APOE is the target gene of IGF2BP2 and FTO-mediated m^6^A modification of APOE is dependent on IGF2BP2.

APOE is a member of apolipoproteins which binds lipids to form lipoproteins and transport them to various body tissues and organs. APOE is comprised of 299 amino acids, and has three main alleles: APOE-ε2, APOE-ε3 and APOE-ε4. Previous studies report that APOE is mainly responsible for metabolism and redistribution of cholesterol, whereas recent studies report that its expression is upregulated and exerts biological functions in various cancers [42]. APOE is significantly highly expressed in lung cancer, and its overexpression promotes cancer proliferation and migration, and aggressiveness of lung cancer [43]. APOE knockout inhibits tumor growth and metastasis by increasing REEM-1-mediated infiltration of NK cells in lung cancer [44]. Notably, APOE modulates brain glucose metabolism in AD [45], and affects glucose uptake in atherosclerosis [46]. These findings indicate that APOE may exert regulatory role in tumor energy metabolism. The findings of the current study showed that the APOE expression was significantly upregulated and positively correlated with tumor size ≥2 cm and extrathyroidal invasion. In addition, APOE knockdown significantly inhibited glycolysis and abrogated its proliferation ability, and could reverse the promotional effect induced by downregulation of FTO in PTC cells. Findings from in vivo experiments showed similar phenomena. These findings indicate that APOE could promote tumor growth through glycolysis in PTC.

Furthermore, GSEA based on the RNA-seq data and TCGA gene expression profiling data showed that IL-6/JAK/STAT3 signaling pathway was highly enriched in si-FTO group and APOE high-expression group. This prediction indicated that the FTO/APOE axis may modulate glycolysis in PTC by regulating IL-6/JAK/STAT3 signaling pathway. IL-6/JAK/STAT3 signaling pathway is abnormally hyperactivated in various cancers, and is associated with poor prognosis. This signaling pathway promotes proliferation, invasiveness, and metastasis of cancer cells, and inhibits anti-tumor immune response [47]. Moreover, recent studies reported that the IL-6/JAK/STAT3 signaling pathway is associated with glycolysis metabolism of cancer cells. LncRNA SLC2A1-AS1 affects aerobic glycolysis and progression by inhibiting STAT3/FOXM1/GLUT1 signaling pathway in hepatocellular carcinoma [48]. In addition, pSTAT3 expression is significantly high in oral squamous cell carcinoma (OSCC), and STAT3 knockdown significantly inhibited migration, invasion, and epithelial-mesenchymal transition (EMT) in OSCC [49]. In the current study, FTO knockdown significantly increased protein expression of pJAK2 and pSTAT3, whereas APOE knockdown reduced expression of the two phosphorylated proteins. This finding indicated that APOE may affect glycolysis of PTC by modulating IL-6/JAK/STAT3 signaling pathway after being modified by FTO. However, the specific mechanism should be further explored. On the other hand, our findings were performed in papillary thyroid cancer tissues and cells, and cannot exclude that the same mechanisms are involved in other histotypes of thyroid tumors or other kinds of tumors. The findings for the current study are summarized in the schematic model shown in Fig. 6h.

## Conclusion

The current study explored the crucial role of m^6^A demethylase FTO and its inhibitory effect on glycolysis in PTC. The findings indicated that FTO epigenetically inhibits expression of APOE through IGF2BP2-mediated m^6^A modification. These changes result in inhibition of glycolytic metabolism of PTC through modulation of the IL-6/JAK2/STAT3 signaling pathway, ultimately affecting tumor growth. These findings show a novel molecular mechanism of PTC progression regulated by m^6^A modification and provide valuable insights for development of efficient therapeutic strategies against PTC.

## Supplementary Information

**Additional file 1: Table S1.** The sequence of si-RNAs. **Table S2.** The sequence of primers. **Table S3.** The Antibodies for western blot, IHC and RIP. **Table S4.** Correlation between FTO expression and clinicopathological features of PTC patients in TCGA database (*n* = 428). **Table S5.** Correlation between APOE expression and clinicopathological features of PTC patients in TCGA database (n = 428).

**Additional file 2.** Supplementary materials and methods.

**Additional file 3: Figure S1.** FTO inhibits cell proliferation in PTC. **a.** Overexpression efficiency of FTO in K1 cells determined by qRT-PCR and western blotting. **b.** Knockdown efficiency of si-FTO-1/2 in TPC1 cells as determined by qRT-PCR and western blotting. **c.** Total m^6^A modification levels after FTO knockdown or overexpression in PTC cells as determined by m^6^A RNA Methylation Quantification Kit. **d-e.** CCK-8 assay (**d**) and colony formation assay (**e**) showing proliferation ability in K1 cells after FTO overexpression. **f-g.** CCK-8 assay (**f**) and colony formation assay (**g**) showing proliferation ability after FTO knockdown in TPC1 cells. **h.** Flow cytometry was performed to measure cell cycle distribution after FTO knockdown in K1 cells. **i.** Flow cytometry was performed to measure cell cycle distribution after FTO overexpression in TPC1 cells. **j.** Knockdown efficiency of sh-FTO in K1 cells determined by western blotting. *P < 0.05, **P < 0.01. **Figure S2.** FTO inhibition of tumor growth by modulating glycolytic metabolism in PTC. **a.** ECAR as determined by Seahorse metabolic analysis after transfection with si-NC and si-FTO in TPC1 cells. **b-c.** Oxygen consumption rate (OCR) as determined by Seahorse metabolic analysis after transfection with si-NC and si-FTO in PTC cells. **d-e.** Glucose uptake (**d**) and Lactate production (**e**) were determined after transfection with si-NC and si-FTO in TPC1 cells. **f.** Protein expression of GLUT1, HK-II and LDHA were determined by western blotting after FTO knockdown in TPC1 cells. **g-h.** CCK-8 assay (**g**) and colony formation assay (**h**) showing proliferation ability after transfection with si-NC or si-FTO and simultaneous treatment with or without 2-DG in TPC1 cells. **i.** ECAR as determined by Seahorse metabolic analysis after FTO overexpression in K1 cells. **j-k.** Glucose uptake (**j**) and Lactate production (**k**) were determined after FTO overexpression in K1 cells. **l-m.** OCR as determined by Seahorse metabolic analysis after FTO overexpression in PTC cells. **n.** GLUT1, HK-II and LDHA protein expression as determined by western blotting after FTO overexpression in K1 cells. *P < 0.05, **P < 0.01. **Figure S3.** APOE mRNA is regulated by FTO-mediated m^6^A modification which is modulated by m^6^A reader IGF2BP2. **a.** Gene ontology (GO) analysis of differentially expressed gene sets in FTO knockdown cells. **b**. Pathway analysis of elevated m^6^A peak-related gene sets in FTO knockdown cells. **c-f.** APOE mRNA and protein expression levels as determined by qRT-PCR and western blotting after FTO overexpression or knockdown in PTC cells. **g.** Relative APOE mRNA expression level as determined by qRT-PCR after transfection with si-NC or si-FTO and simultaneous treatment with or without DAA in TPC1 cells. **h-i.** Relative luciferase activity of APOE-Wt and APOE-Mut after FTO overexpression or knockdown in PTC cells. **j.** Half-life (t_1/2_) of APOE mRNA as determined by qRT-PCR after FTO knockdown in TPC1 cells. **k.** Relative expression level of APOE mRNA as determined by qRT-PCR after IGF2BP1–3 knockdown in TPC1 cells. **l.** Half-life (t_1/2_) of APOE mRNA after IGF2BP2 knockdown as determined by qRT-PCR in TPC1 cells. **Figure S4.** APOE is negatively regulated by FTO and modulates glycolysis to regulate PTC proliferation. **a-d.** Knockdown efficiency of si-APOE-1/2 as determined by qRT-PCR and western blotting in PTC cells. **e-f.** CCK-8 assay (**e**) and colony formation assay (**f**) showing proliferation ability after transfection with si-FTO or co-transfection with si-FTO and si-APOE in TPC1 cells. **g.** ECAR as determined by Seahorse metabolic analysis after transfection with si-FTO or co-transfection with si-FTO and si-APOE in TPC1 cells. **h-i.** OCR as determined by Seahorse metabolic analysis after transfection with si-FTO or co-transfection with si-FTO and si-APOE in PTC cells. **j-k.** Glucose uptake (**j**) and Lactate production (**k**) after transfection with si-FTO or co-transfection with si-FTO and si-APOE in TPC1 cells. **l.** Protein expression level of GLUT1, HK-II and LDHA as determined by western blotting after APOE knockdown in TPC1 cells. **m.** CCK-8 assay showing proliferation ability after transfection with empty vector or APOE and simultaneous treatment with or without 2-Deoxyglucose (2-DG) in TPC1 cells. **n-o.** Colony formation assay showing proliferation ability after transfection with empty vector or APOE and simultaneous treatment with or without 2-Deoxyglucose (2-DG) in PTC cells. **Figure S5.** FTO and APOE regulate glycolysis of PTC by modulating IL-6/JAK2/STAT3 signaling pathway. **a.** Differential gene profiles based on GSEA of APOE gene expression profile data of TCGA. **b.** Expression level of IL-6/JAK2/STAT3 signaling pathway related proteins as determined by western blotting after FTO knockdown in TPC1 cells. **c.** Expression levels of IL-6/JAK2/STAT3 signaling pathway related proteins as determined by western blotting after FTO overexpression in K1 cells. **d.** Expression level of IL-6/JAK2/STAT3 signaling pathway related proteins as determined by western blotting after APOE knockdown in TPC1 cells. **e.** Expression levels of IL-6/JAK2/STAT3 signaling pathway related proteins as determined by western blotting after APOE overexpression in K1 cells. **f.** Expression level of GLUT1 protein and IL-6/JAK2/STAT3 signaling pathway related proteins as determined by western blotting after co-transfection with si-FTO and si-APOE in TPC1 cells.

## Data Availability

The data used during the current study are available from the corresponding author on reasonable request. The RNA-seq and MeRIP-seq data discussed in this publication have been deposited in NCBI’s Gene Expression Omnibus and are accessible through GEO Series accession number GSE181047 (https://www.ncbi.nlm.nih.gov/geo/query/acc.cgi?acc=GSE181047).

## Abbreviations

2-DG: 2-deoxy-glucose
AD: Alzheimerʼs disease
ALKBH5: Alkylation repair homolog protein 5
APOE: Apolipoprotein E
BAG3: Bcl-2-associated athanogene 3
DAA: 3-Deazaadenosine
DEGs: Differentially expressed genes
DTC: Differentiated thyroid cancer
ECAR: Extra cellular acidification rate
EMT: Epithelial-mesenchymal transition
FDR: False discovery rate
^18^F-FDG PET: ^18^F-fluoro-2-deoxyglucose positron emission tomography
FFPE: Formalin-fixed and paraffin-embedded
FTO: Fat-mass and obesity-associated protein
GEO: Gene Expression Omnibus
GO: Gene ontology
GSEA: Gene set enrichment analysis
HK-II: Hexokinase II
IGF2BP: Insulin-like growth factor 2 mRNA-binding proteins
IGV: Integrative Genomics Viewer
IHC: Immunohistochemistry
m^6^A: N6-methyladenosine
MeRIP-seq: Methylated RNA immunoprecipitation sequencing
METTL3: Methyltransferase-like 3
METTL14: Methyltransferase-like 14
mRNA: Messenger RNA
MTA1: Metastasis-associated protein 1
NES: Normalized enrichment score
OCR: Oxygen consumption rate
OSCC: Oral squamous cell carcinoma
pJAK2: Phosphorylated JAK2
pSTAT3: Phosphorylated STAT3
PTC: Papillary thyroid carcinoma
qRT-PCR: Quantitative real-time PCR
RIP: RNA immunoprecipitation
RNA-seq: Transcriptome-sequencing
ROI: Region of interest
SD: Standard deviation
siRNA: Short interfering RNA
SUVmax: Maximum standardized uptake values
TCGA: The Cancer Genome Atlas
WTAP: Wilms tumor 1 associated protein
YTHDC: YTH domain-containing proteins
YTHDF: YTH domain family proteins
