# Supplementary material for: FTO suppresses glycolysis and growth of papillary thyroid cancer via decreasing stability of APOE mRNA in an N6-methyladenosine-dependent manner
Source: J Exp Clin Cancer Res. 2022 Jan 28;41:42. doi: 10.1186/s13046-022-02254-z (PMC8796435; doi:10.1186/s13046-022-02254-z)
Supplement: Supplementary file 2 — Additional file 2. Supplementary materials and methods. [file 13046_2022_2254_MOESM2_ESM.pdf]

Supplementary materials and methods

Cell culture

Nthy-ori3-1 and K1 cell lines were purchased from the European Collection of Authenticated Cell Culture (ECACC, UK). BCPAP cell line was obtained from DSMZ (Braunschweig, Germany). IHH4 cell line was obtained from the Health Science Research Resources Bank (Osaka, Japan). TPC1 cell line was a kind gift from Professor Meiping Shen (Department of General Surgery, The First Affiliated Hospital of Nanjing Medical University, Nanjing, Jiangsu). BCPAP cell line and Nthy-ori3-1 cell line were cultured in Roswell Park Memorial Institute (RPMI)-1640 medium supplemented with 10% fetal bovine serum (FBS). K1 cell line was maintained in Dulbecco's modified eagle's medium (DMEM): Ham's F12: MCDB 105 (2:1:1) supplemented with 10% FBS. IHH4 cell line was maintained in RPMI-1640 and DMEM (1:1) supplemented with 10% FBS. TPC1 cells were maintained in DMEM with 10% FBS. All cell lines were incubated under an atmosphere of 5% CO_2 at 37℃

Total RNA extraction and qRT-PCR

Total RNA was extracted from frozen specimens obtained from tissue samples and cells using RNAiso Plus (Takara, Japan). Reverse Transcription was performed using PrimeScript™ RT Master Mix. qRT-PCR was performed using SYBR Premix Ex Taq II (Takara, Japan) on a Light Cycle0r 480 system (Roche, USA). Primer sequences used in the current study are shown in Additional file 1: Table S1. The $2^{-\Delta\Delta Ct}$ method was used to calculate the relative expression levels (CT, cycle threshold). GAPDH

was used as an internal control.

Western Blotting

Total proteins were extracted from PTC cells and tissue samples using Protein Extraction Kit (KeyGEN, China). 40-50ug of proteins were subjected to 10% sodium dodecyl sulfate polyacrylamide gel electrophoresis (SDS-PAGE), and the separated proteins were then transferred to polyvinylidene difluoride membranes (Millipore, Billerica, MA, USA). The membranes were blocked with 5% skim milk for 2h, and then incubated with primary antibodies diluted with primary antibody dilution buffer overnight at 4℃. Details on primary antibodies are presented in Additional file 1: Table S3. The membranes bound with antibodies were incubated with secondary antibodies (1:1,000 dilution, ZSGB-Bio, China). Protein bands were visualized on MicroChemi imaging system using an enhanced chemiluminescence (ECL) kit (Thermo, USA).

Immunohistochemistry (IHC)

PTC tissue sections were baked at 65℃ for 30min, then rehydrated with a series of alcohol concentrations. Endogenous peroxidase activity was blocked with hydrogen peroxide. Sections were treated by microwave with citrate buffer and incubated with anti-FTO and anti-APOE on a humidified box overnight at 4℃. Immunohistochemical staining score was determined using semi-quantitative Remmele scoring system.

Total m6A level assays

Total RNA was extracted from tissues and cells using RNAiso Plus (Takara, Japan).

Total m6A level was determined using EpiQuik™ m^6A RNA Methylation Quantification Kit (Colorimetric, Epigentek, USA) according to the manufacturer's protocol. The optimal quantification of the input RNA was 200ng.

Cell proliferation and colony formation Assay

Cell proliferation rate was determined by Cell Counting Kit-8 (CCK-8; Dojindo, Japan). K1 and TPC1 were seeded into 96-well plates, and then transfected with si-NC, si-FTO or si-APOE. Cell lines with stably overexpressed FTO or APOE and empty vector were directly seeded into 96-well plates. CCK-8 solution was added to each well at 0, 24, 48, and 72h after gene transfection, and then absorbance was determined at 450nm wavelength after incubation for 2-4h.

For the Colony formation assay, transfected or Lentivirus infected cells were counted and 500 cells were seeded per well into 6-well plates. Cells were incubated for 7 to 10 days, then fixed with 4% paraformaldehyde and then stained with crystal violet. After washing and drying, cell colonies were photographed and counted.

Cell cycle analysis

The transfected or Lentivirus infected cells were adjusted to the same cell concentration and fixed in 75% ethanol for 2 h at 4℃. The fixation solution was then washed with PBS and stained with 500 μL propidium iodide/RNase A (1:9) for 30 min at room temperature. Finally, the samples were analyzed using flow cytometry with CellQuest 3.0 software.

RNA stability assay

si-NC, si-FTO or si-IGF2BP2 transfected PTC cells were treated with Actinomycin D,

and total RNA was extracted at 0h, 3h and 6h after treatment. Relative expression of APOE mRNA was then determined by qRT-PCR after quantitation and reverse transcription. Degradation rate and half-life of APOE mRNA were calculated by nonlinear regression.

Bioinformatic analyses

Raw gene expression data of m6A modification enzymes in PTC were retrieved from TCGA database and GEO database. We initially obtained gene expression profiles of 510 PTC tissues and 58 normal thyroid tissues from TCGA database. After averaging gene expression values of the same cases, the expression profiles of 502 PTC tissues were left. The expression of m6A modification enzymes in normal thyroid tissues and PTC tissues were analyzed. Then, corresponding clinicopathological data of the remaining 502 patients were matched by TCGA database. After excluding cases with non-PTC pathological types and those with incomplete clinicopathological data such as unknown tumor size, unknown lymph nodes and unknown distant metastasis, the correlation between gene expression and clinicopathological data was analyzed for the remaining 428 cases. Gene set enrichment analysis (GSEA) was performed to identify significantly enriched signaling pathways using RNA-seq data and data on TCGA gene expression. Normalized enrichment score (NES) and false discovery rate (FDR) were used to determine statistical difference.
