# Supplementary figures and images for: FTO suppresses glycolysis and growth of papillary thyroid cancer via decreasing stability of APOE mRNA in an N6-methyladenosine-dependent manner
Source: J Exp Clin Cancer Res. 2022 Jan 28;41:42. doi: 10.1186/s13046-022-02254-z (PMC8796435; doi:10.1186/s13046-022-02254-z)

**Figure S1**

**a**

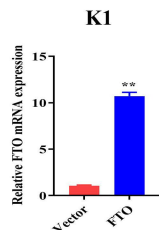

**b**

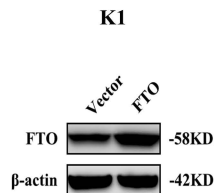

**TPC1**

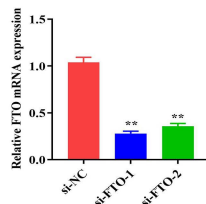

**TPC1**

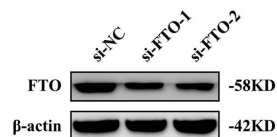

**c**

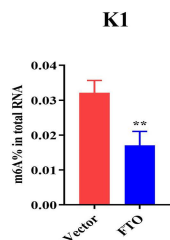

**TPC1**

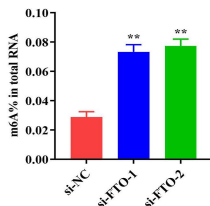

**d**

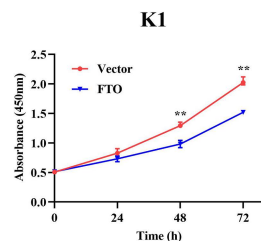

**e**

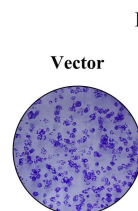

**K1**

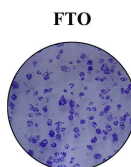

**K1**

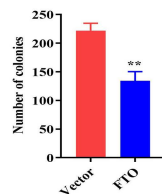

**f**

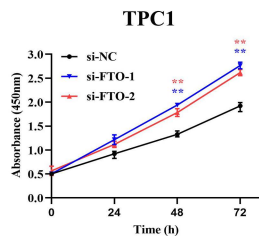

**g**

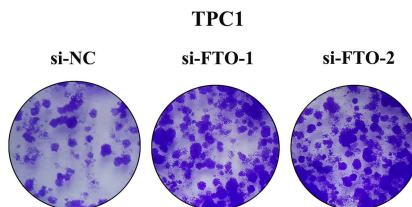

**TPC1**

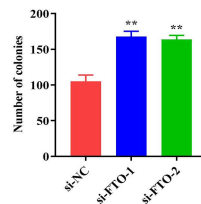

**h**

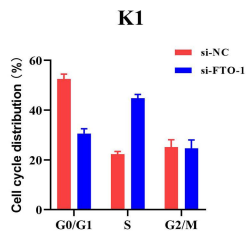

**i**

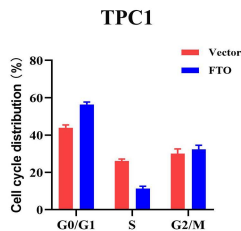

**j**

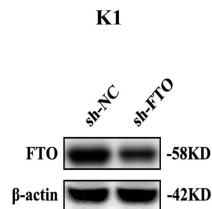

**Figure S2**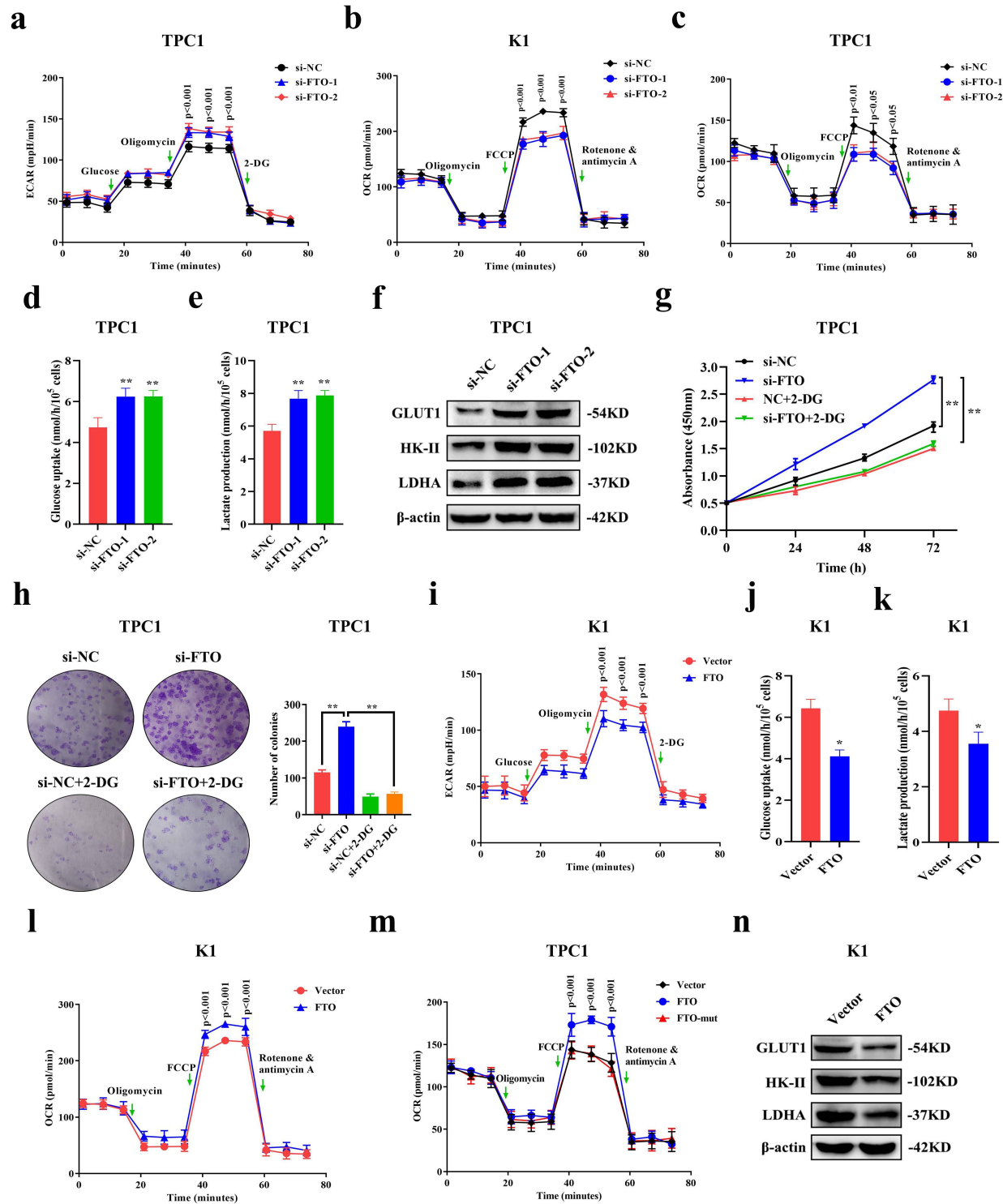

# Figure S3

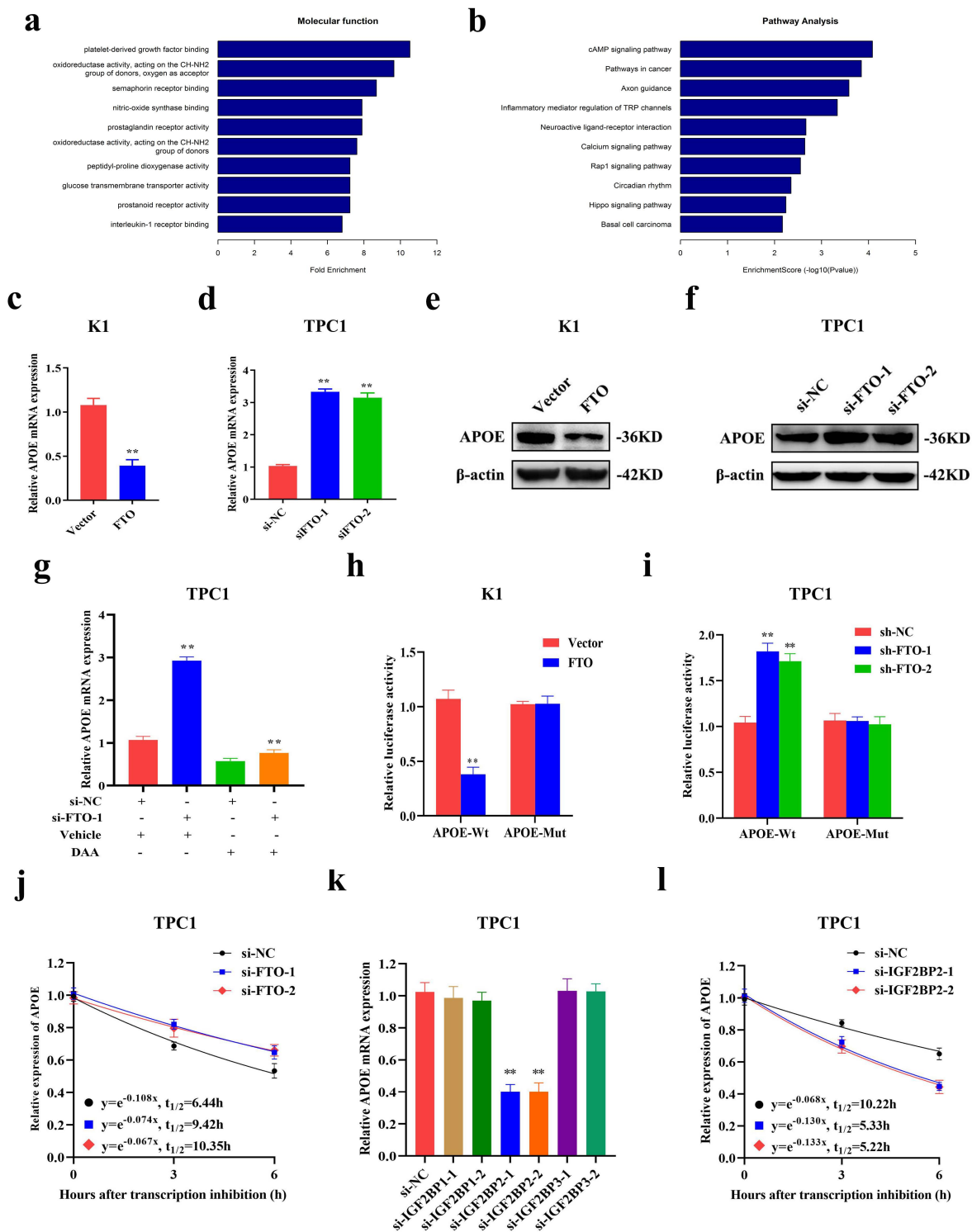

**Figure S4**

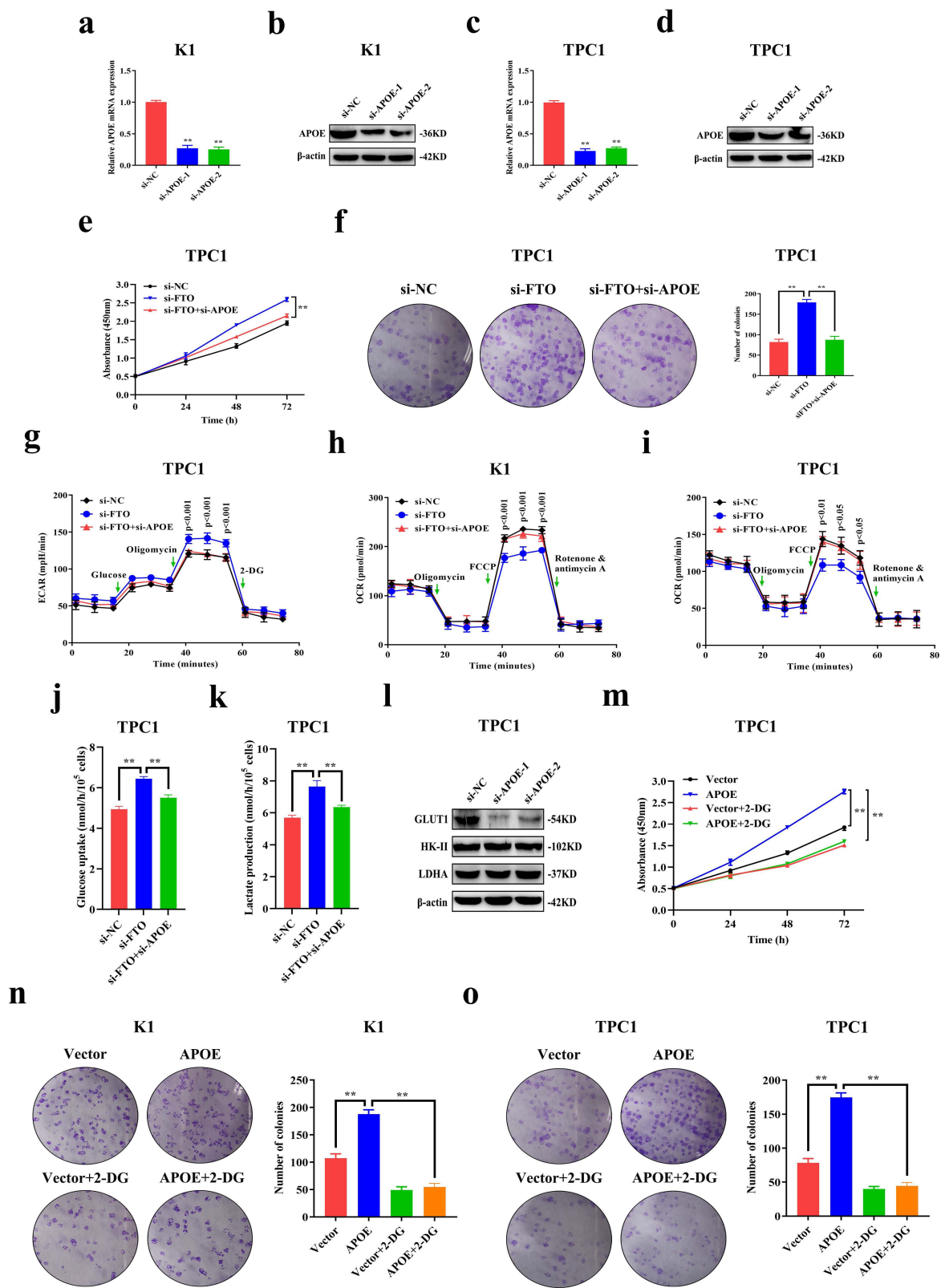

# Figure S5

**a**

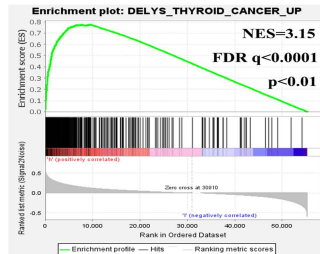

**b**

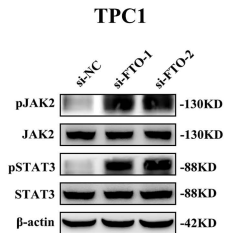

**f**

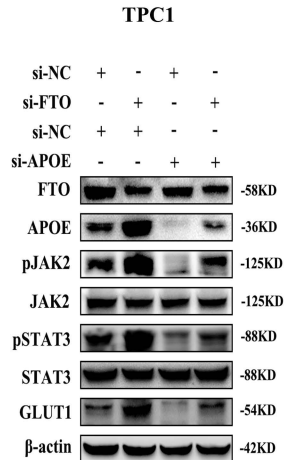

**c**

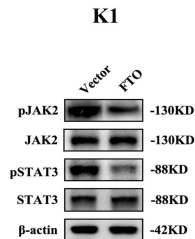

**d**

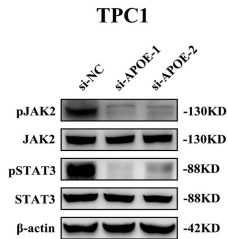

**e**

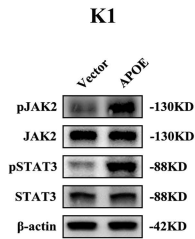

Supplement: Supplementary file 3 — Additional file 3: Figure S1. FTO inhibits cell proliferation in PTC. a. Overexpression efficiency of FTO in K1 cells determined by qRT-PCR and western blotting. b. Knockdown efficiency of si-FTO-1/2 in TPC1 cells as determined by qRT-PCR and western blotting. c. Total m6A modification levels after FTO knockdown or overexpression in PTC cells as determined by m6A RNA Methylation Quantification Kit. d-e. CCK-8 assay (d) and colony formation assay (e) showing proliferation ability in K1 cells after FTO overexpression. f-g. CCK-8 assay (f) and colony formation assay (g) showing proliferation ability after FTO knockdown in TPC1 cells. h. Flow cytometry was performed to measure cell cycle distribution after FTO knockdown in K1 cells. i. Flow cytometry was performed to measure cell cycle distribution after FTO overexpression in TPC1 cells. j. Knockdown efficiency of sh-FTO in K1 cells determined by western blotting. *P < 0.05, **P < 0.01. Figure S2. FTO inhibition of tumor growth by modulating glycolytic metabolism in PTC. a. ECAR as determined by Seahorse metabolic analysis after transfection with si-NC and si-FTO in TPC1 cells. b-c. Oxygen consumption rate (OCR) as determined by Seahorse metabolic analysis after transfection with si-NC and si-FTO in PTC cells. d-e. Glucose uptake (d) and Lactate production (e) were determined after transfection with si-NC and si-FTO in TPC1 cells. f. Protein expression of GLUT1, HK-II and LDHA were determined by western blotting after FTO knockdown in TPC1 cells. g-h. CCK-8 assay (g) and colony formation assay (h) showing proliferation ability after transfection with si-NC or si-FTO and simultaneous treatment with or without 2-DG in TPC1 cells. i. ECAR as determined by Seahorse metabolic analysis after FTO overexpression in K1 cells. j-k. Glucose uptake (j) and Lactate production (k) were determined after FTO overexpression in K1 cells. l-m. OCR as determined by Seahorse metabolic analysis after FTO overexpression in PTC cells. n. GLUT1 [file 13046_2022_2254_MOESM3_ESM.pdf]
